# Recent advances in transition-metal-catalyzed incorporation of fluorine-containing groups

**DOI:** 10.3762/bjoc.15.218

**Published:** 2019-09-23

**Authors:** Xiaowei Li, Xiaolin Shi, Xiangqian Li, Dayong Shi

**Affiliations:** 1 Key Laboratory of Experimental Marine Biology, Institute of Oceanology, Chinese Academy of Sciences, 7 Nanhai Road, Qingdao 266071, China; 2 Laboratory for Marine Drugs and Bioproducts of Qingdao National Laboratory for Marine Science and Technology, Qingdao 266000, China; 3 University of Chinese Academy of Sciences, Beijing 100049, China; 4 State Key Laboratory of Microbial Technology, Shandong University, 72 Binhai Road, Qingdao 266237, China

**Keywords:** catalysis, C-R_F_ bond, fluorination, fluoroalkylation, transition metals

## Abstract

Fluorine chemistry plays an increasingly important role in pharmaceutical, agricultural, and materials industries. The incorporation of fluorine-containing groups into organic molecules can improve their chemical and physical properties, which attracts continuous interest in organic synthesis. Among various reported methods, transition-metal-catalyzed fluorination/fluoroalkylation has emerged as a powerful method for the construction of these compounds. This review attempts to describe the major advances in the transition-metal-catalyzed incorporation of fluorine, trifluoromethyl, difluoromethyl, trifluoromethylthio, and trifluoromethoxy groups reported between 2011 and 2019.

## Introduction

Compared with other halogens (Cl, Br, I), fluorine (F) has completely different physical and chemical properties, such as a unique electronic structure, strongest electronegativity, and small atomic radius similar to that of hydrogen atoms. Due to these unique properties, the introduction of fluorine into a molecule can cause dramatic changes, such as the acidity or basicity of neighboring groups, dipole moment, and properties such as lipophilicity, metabolic stability, and bioavailability [1]. Consequently, carbon–fluorine bonds have become an integral part of pharmaceutical [2–3], agricultural [4], materials industries [5], and tracers for positron emission tomography [6]. According to statistics, about 35% of agrochemicals and 20% of pharmaceuticals contain fluorine [7].

Although the content of fluorine in the Earth’s crust is relatively abundant (13th most abundant element), scientists have identified only 21 kinds of fluorine-containing natural molecules [8–9]. Therefore, it is highly desirable to introduce a fluorine-containing substituent into a molecule artificially. However, traditional fluorination methods to these building blocks, such as Friedel–Crafts-type electrophilic halogenation [10–11], Sandmeyer-type reactions of diazonium salts [12], and halogenations of preformed organometallic reagents [13], commonly involve multiple steps, harsh reaction conditions, and the use of stoichiometric amounts and/or toxic reagents [14]. Also, low functional group tolerance, being limited to activated arenes, the production of metal salts as stoichiometric byproducts, and poor levels of regioselectivity would always be observed, limiting the progress of fluorine chemistry to some extent. In this regard, the use of various transition metals to catalyze the synthesis of organic fluorides has become a mature field, and the application of these methodologies has allowed decreasing the need of pre-functionalized substrates, less consumption of reaction time and costs, and enabled to produce enantioenriched target compounds [15–20]. Furthermore, transition metals have the unique advantage of possessing multiple mechanistic features, which translates into the ability to apply new substrate classes and provide hitherto novel and inaccessible structures. Therefore, transition-metal-catalyzed fluorination/fluoroalkylation reactions represent an important and hot topic in fluorine chemistry. In addition, among the various metals developed, palladium is the most commonly employed transition metal, followed by copper owing to its high efficiency and cheapness. Meanwhile, other transition metals, such as Fe, Ni, Rh, Ag, Co, etc., have received considerable attention and are widely applied due to their respective characteristics.

Over the past few years, several reviews on fluorination/fluoroalkylation have disclosed. Kamlet [17] mainly discussed progresses in catalyzed fluorination and trifluoromethylation before 2011, and Besset [21] focused on the direct introduction of fluorinated groups into alkenes and alkynes. Then, Toste [1] covered advances in catalytic enantioselective fluorination, mono‑, di‑, and trifluoromethylation, and trifluoromethylthiolation reactions. Recently, Zhang [14] offered a brief summary of the recent achievements in the ever-growing field of green fluoroalkylation. However, until now, no comprehensive survey of the literature has been reported on this topic. In this review, we highlight the recent progress of transition-metal-catalyzed fluorination and trifluoromethylation reported between 2011 and 2019. Meanwhile, we also present the incorporation of difluoromethyl, trifluoromethylthiol and trifluoromethoxy groups. Some sections of this review are structured around the synthesis of alkyl-, aryl- and vinyl- as well as alkynyl organofluorides. Notably, the current review covers mainly two types of transition-metal-catalyzed reactions: 1) cross-couplings with a fluorinated organometallic species or a halogenated fluorinated species and 2) the direct introduction of fluorinated moieties into nonfunctionalized substrates with a fluorinated reagent. We hope that this review will provide a comprehensive overview of this topic and attract significant attention.

## Review

### Fluorination

**Scheme 1:**
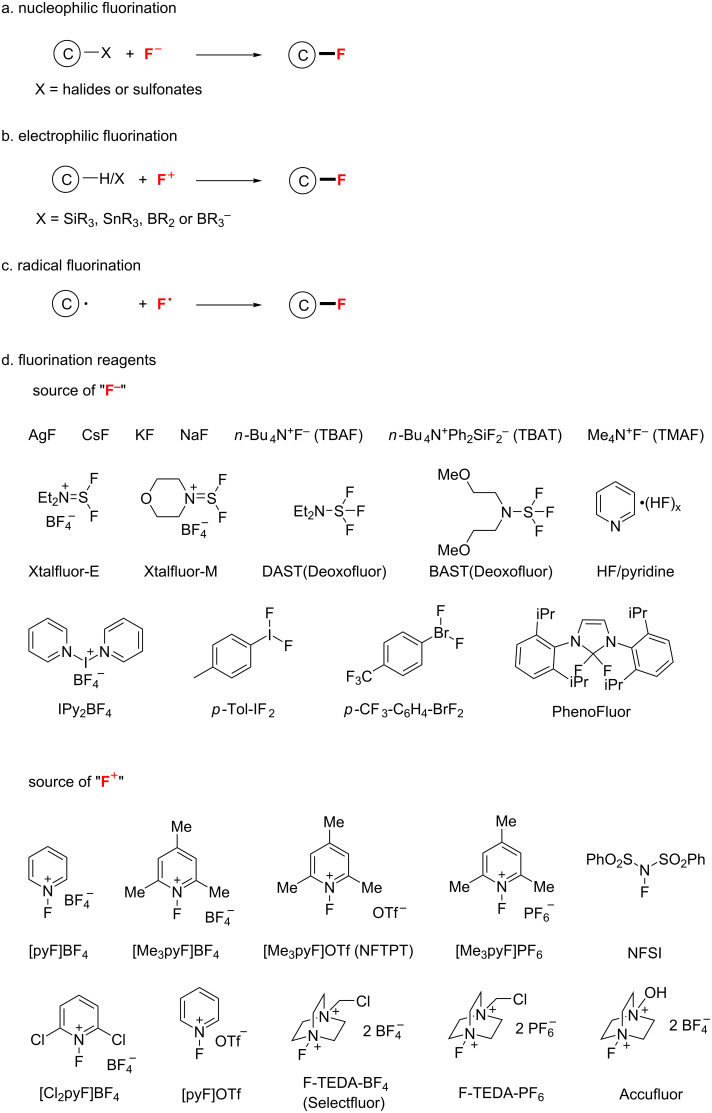
The main three strategies of fluorination: nucleophilic, electrophilic and radical fluorination.

For many years, specialists in the field of fluorine chemistry have been actively studying ways to introduce fluorine into organic molecules by aid of transition-metal catalysis. Depending on the transfer form of fluorine, there are three general strategies for constructing C–F bonds: nucleophilic, electrophilic and radical fluorination (Scheme 1) [22].

In nucleophilic fluorination reactions, the fluoride anion (F^−^) or a derivative thereof, such as tetrafluoroborate (BF_4_^−^), is the fluorine source and behaves as a nucleophile. The electrophile, such as an alkyl chain or an aryl ring with halides or sulfonates, reacts with the fluoride source (Scheme 1a). On the other hand, in the electrophilic fluorination, the nucleophile may be a carbon anion (e.g., Grignard reagent), a compound with electron-rich unsaturated bonds (arene, alkene, or alkyne), or a substrate having a nucleophilic and labile bond (e.g., C−Si, C−Sn, and C−B), while the electrophile is the fluorination reagent (Scheme 1b). As shown in Scheme 1d, many nucleophilic and electrophilic fluorination reagents have been developed by chemists. In the radical fluorination, C–F bonds are produced by carbon-based radicals (generated in situ by various methods) with "atomic fluorine" sources, such as XeF_2_, hypofluorite, or molecular fluorine (Scheme 1c). Notably, transition metals are not biased to one reaction class, and the same metal may be successfully applied to all three kinds of fluorination.

Several reviews of fluorination have been published within the past few years, Buchwald [23], Weng [24], Gouverneur [25], Reiser [26], and etc. [22,27–33] discussed progresses of fluorination, such as Weng who focused on the recent advances in the transition-metal-assisted synthesis of alkyl fluorides, and Buchwald introduced the discovery and development of Pd(0)/Pd(II)-catalyzed aromatic fluorination reactions. Herein, we focus on the developments towards the construction of C(sp^3^)–F and C(sp^2^)–F bonds with different catalysts, such as palladium, copper, silver, iron, nickel, ruthenium, cobalt, etc.

#### Palladium catalysis

Palladium is a member of the nickel triad in the periodic table, and palladium complexes exist in three oxidation states, Pd(0), Pd(II), and Pd(IV). Straightforward interconversion between different oxidation states, tolerance to various guiding groups, easy electroplating of C–H bonds, and the compatibility of many Pd(II) catalysts with oxidants make them act as ideal catalysts for C–H activations [34]. Over the last decade, a number of Pd-catalyzed methods have been developed to synthesize aryl fluorides [23,32].

**Scheme 2:**
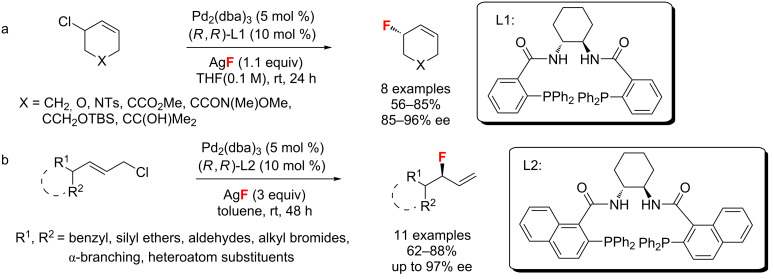
Doyle’s Pd-catalyzed fluorination of allylic chlorides.

**Allylic fluorination:** In 2010, Doyle and co-worker [35] developed a strategy for C–F bond formation of readily available cyclic allylic chlorides and AgF using a Pd(0) catalyst in combination with Trost’s bisphosphine ligand at room temperature (Scheme 2a). They also proved that the allylic fluorination was achieved by an S_N_2-type attack of fluoride on an electrophilic Pd(II)-allyl intermediate. One year later, the same author extended this method to a highly regio- and enantioselective fluorination of acyclic allylic chlorides. Compared to the previous process, this reaction used a different chiral bisphosphine ligand resulting in larger bite angles and afforded the products in good yields (Scheme 2b) [36].

**Scheme 3:**
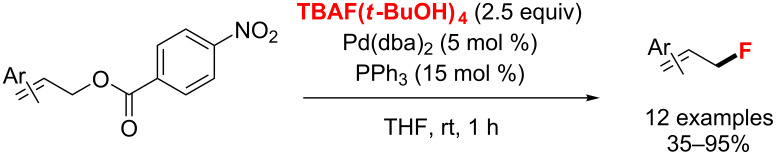
Allylic fluorination of 2- and 3-substituted propenyl esters.

A palladium-catalyzed method for the formation of allylic C–F bonds from allyl *p*-nitrobenzoate using TBAF(*t*-BuOH)_4_ as the fluoride source was explored by Gouverneur et al. in the same year (Scheme 3) [37]. The 2- and 3-arylpropenyl fluorides can be quickly synthesized under mild conditions in moderate to good yields.

**Scheme 4:**
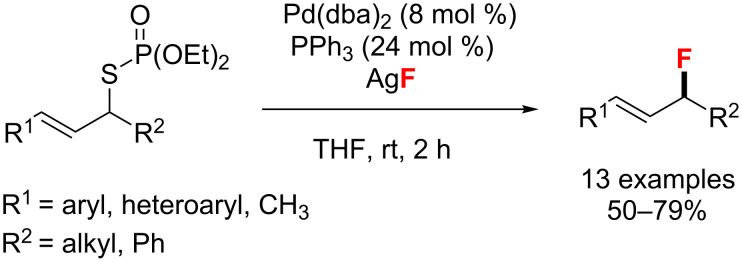
Regioselective allylic fluorination of cinnamyl phosphorothioate esters.

In 2012, a Pd(0)-catalyzed allylic fluorination of allylic phosphorothioate esters with AgF was accomplished by Wu’s group (Scheme 4) [38]. The formation of fluorinated products with an overall retention of the stereochemical configuration suggests a mechanism wherein a palladium-π-allyl intermediate undergoes a rapid π-σ-π isomerization.

**Scheme 5:**
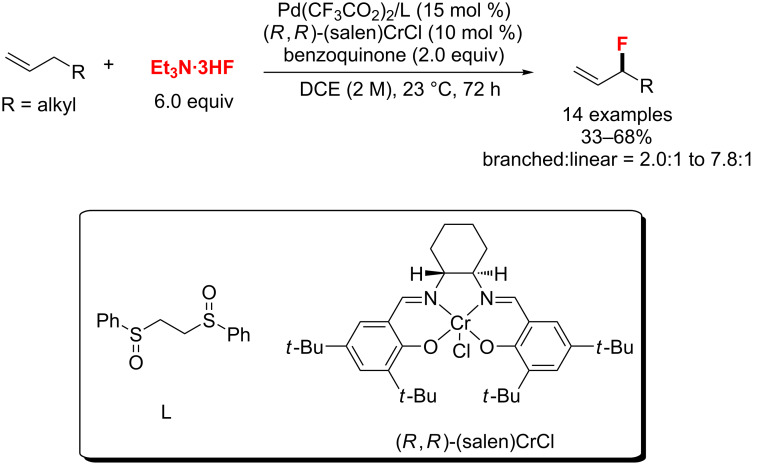
Palladium-catalyzed aliphatic C–H fluorination reported by Doyle.

In 2013, the first example of an allylic C–H fluorination reaction of simple alkenes with Et_3_N·3HF as a nucleophilic fluoride source was reported by Doyle and co-worker (Scheme 5) [39]. Herein, the authors utilized a Pd/Cr cocatalytic system to generate the allylic fluorides with high regioselectivity (branched > linear).

**Scheme 6:**
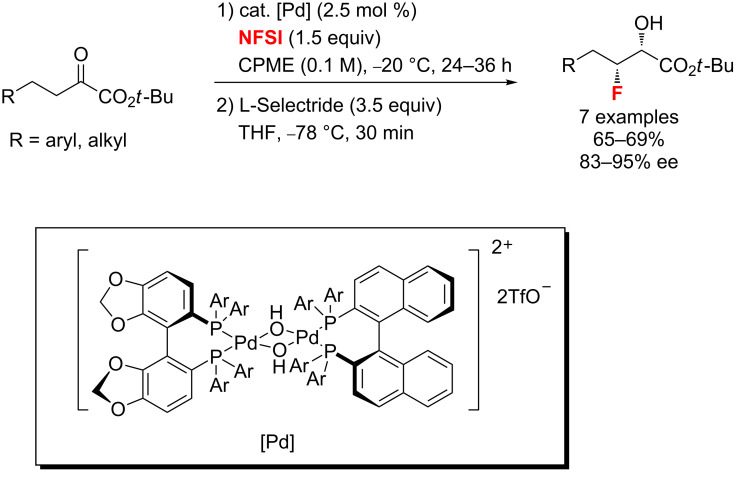
Pd-catalyzed enantioselective fluorination of α-ketoesters followed by stereoselective reduction to give β-fluoro-α-hydroxy esters.

**Alkyl fluorination of acidic carbonyl compounds and other compounds:** In 2012, the group of Sodeoka [40] reported the first example of an enantioselective monofluorination of α-keto esters catalyzed by Pd-μ-hydroxo complexes with cyclopentyl methyl ether (CPME) as the best solvent (Scheme 6). Also, they achieved the diastereoselective reduction of the remaining keto group with lithium tri(*sec*-butyl)borohydride (ʟ-Selectride). The *syn*-β-fluoro-α-hydroxy esters were obtained finally in good yields with excellent enantioselectivities (83–95% ee).

**Scheme 7:**
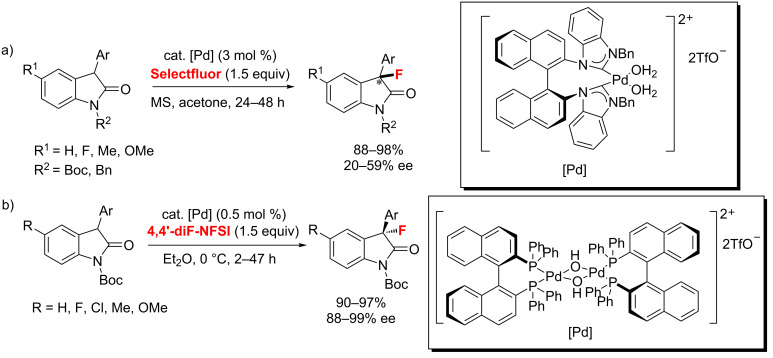
Pd-catalyzed C(sp^3^)–H fluorination of oxindoles.

There are two examples of a Pd-catalyzed fluorination of oxindoles. In 2012, Shi and co-workers [41] described the enantioselective asymmetric fluorination of oxindoles with an axially chiral C_2_-symmetric *N*-heterocyclic carbene (NHC) palladium complex as a catalyst (Scheme 7a). The corresponding products were obtained in excellent yields but low to moderate enantioselectivities. Meanwhile, Wu and co-workers [42] developed a similar system using a BINAP-derived palladium complex to perform the similar reaction with 4,4’-diF-NFSI as the fluorinating agent in higher enantioselectivities (Scheme 7b).

**Scheme 8:**
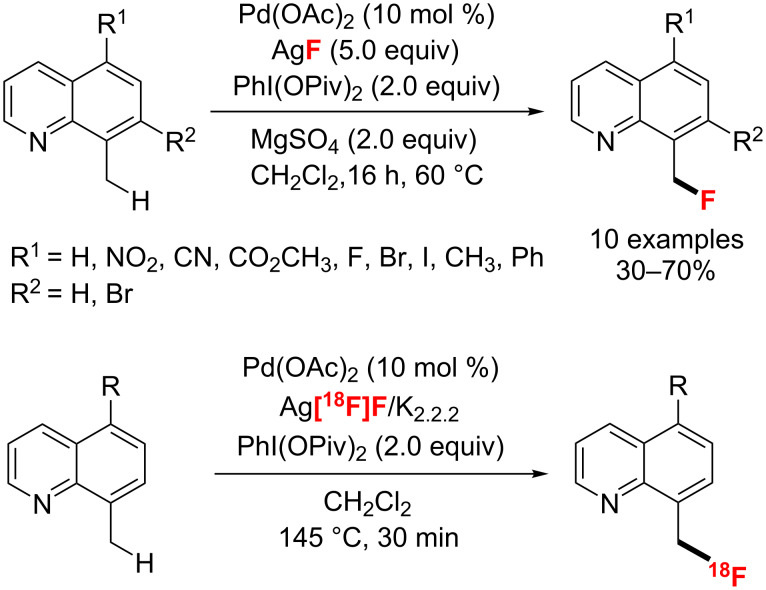
C–H fluorination of 8-methylquinoline derivatives with F^−^ reagents.

In 2012 the group of Sanford [43] achieved the palladium-catalyzed C–H fluorination of 8-methylquinoline derivatives using AgF as the nucleophilic fluoride source and PhI(OPiv)_2_ as a hypervalent iodine oxidant (Scheme 8). Very recently, they [44] optimized this transformation and achieved the benzylic C–H radiofluorination with no-carrier-added Ag[^18^F]F. This method was applied to the radiolabeling of diversely substituted 8-methylquinoline derivatives. Notably, in this process, a new method was developed for generating Ag[^18^F]F by using a sep-pak cartridge.

**Scheme 9:**
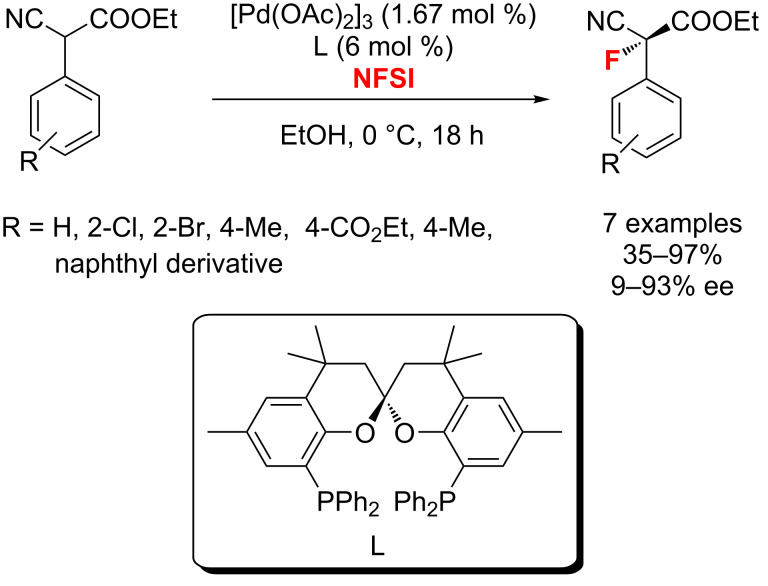
Fluorination of α-cyano acetates reported by van Leeuwen.

In 2012, van Leeuwen and co-workers [45] described the synthesis of new enantiopure wide-bite-angle diphosphanes and their application in the asymmetric fluorination of α-cyanoacetates with a palladium catalyst (Scheme 9). Under these conditions, the fluorination of ethyl 2-cyano-2-phenylacetate afforded the product with highest enantiomeric excess (93%).

**Scheme 10:**
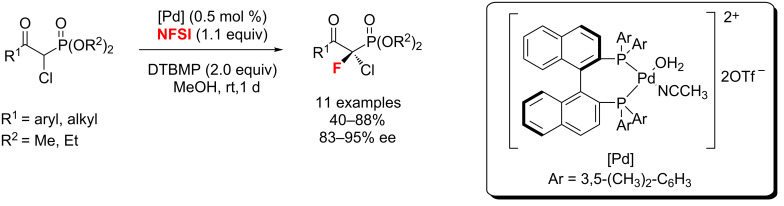
The catalytic enantioselective electrophilic C–H fluorination of α-chloro-β-keto phosphonates.

In 2013, Kim’s group [46] described an enantioselective electrophilic fluorination of α-chloro-β-keto phosphonates with up to 95% ee (Scheme 10). Notably, this reaction used an air and moisture-stable chiral palladium complex as the catalyst, which worked well at low catalyst loading (as low as 0.5 mol %).

**Scheme 11:**
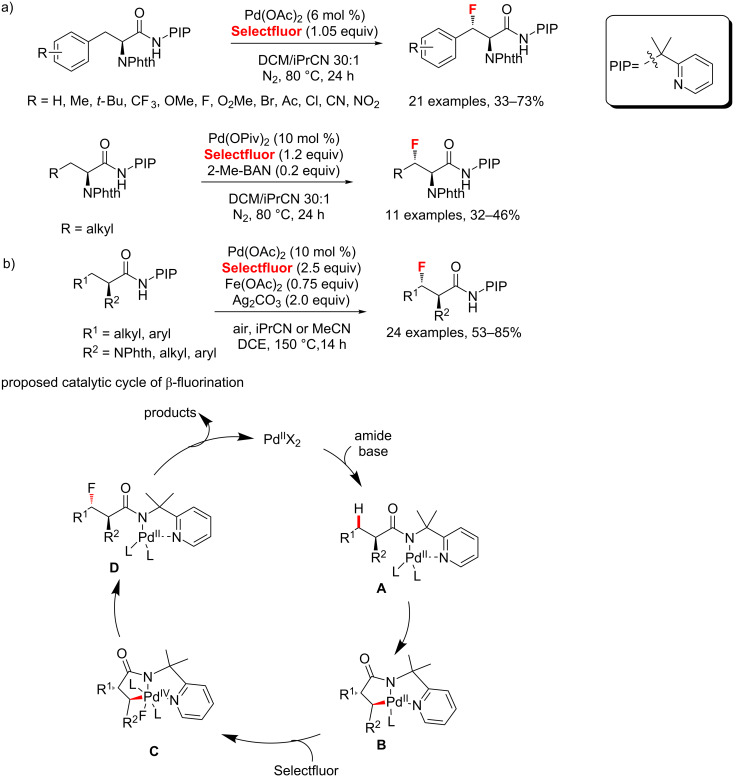
Fluorination of unactivated C(sp^3^)–H bonds directed by the bidentate PIP auxiliary.

In 2015, Shi et al. [47] introduced a Pd(II)/Pd(IV)-catalyzed fluorination of β-methylene C(sp^3^)–H bonds of α-amino acid derivatives (Scheme 11a). This process was carried out under the strongly binding bidentate 2-(pyridine-2-yl)isopropylamine (PIP) auxiliary. A range of substrates containing both aliphatic and benzylic C(sp^3^)–H bonds was finally converted to the corresponding fluorinated products with excellent diastereoselectivities. Based on the PIP auxiliary developed by Shi, Ge’s group [48] developed a similar direct, highly site- and diastereoselective fluorination of aliphatic amides (Scheme 11b). Although the roles of Fe(OAc)_2_ and Ag_2_CO_3_ were unclear, their addition significantly improved the reaction yield. A catalytic cycle of these β-fluorination reactions is proposed in Scheme 11. Initially, coordination of the amide with the palladium species followed by a base-promoted ligand-exchange process yields the palladium complex **A**. Subsequently, cyclometallation of the palladium complex **A** occurs to produce the intermediate **B** through the C–H bond-activation process. Oxidative addition of the intermediate **B** with Selectfluor affords the palladium(IV) species **C**, followed by reductive elimination and ligand dissociation to give the final product.

**Scheme 12:**
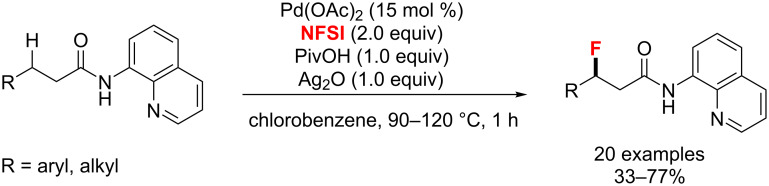
Fluorination of C(sp^3^)–H bonds at the β-position of carboxylic acids.

Similar to these publications in strategy and products, in the same year, Xu’s group [49] presented the palladium-catalyzed direct fluorination of unactivated C(sp^3^)–H bonds at the β-position of carboxylic acids with NFSI (Scheme 12). To achieve this transformation, an 8-aminoquinoline-derived auxiliary was developed as an effective directing group for the activation of the C–H bonds. In this transformation the presence of Ag_2_O and pivalic acid was found to be crucial for the successful synthesis of β-fluorinated carboxylic acids.

**Scheme 13:**
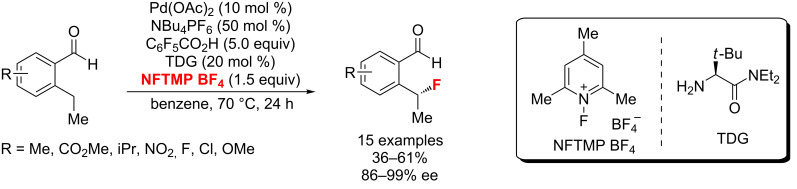
Enantioselective benzylic C–H fluorination with a chiral transient directing group.

Recently, the first example of a Pd-catalyzed protocol for the general enantioselective electrophilic C(sp^3^)–H fluorination of benzaldehyde substrates was reported by Yu and co-workers (Scheme 13) [50]. Enantioenriched benzyl fluorides were obtained by aid of a chiral α-amino amide transient directing group (TDG). Notably, the condensation of this bulky amino amide with the aldehyde led to control of the stereochemistry of the C–H insertion step, promoting the C–F over C–O bond formation via an inner-sphere pathway.

**Scheme 14:**
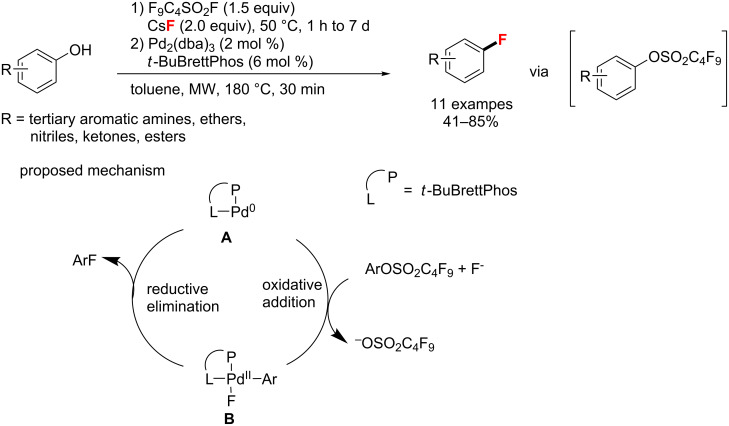
Microwave-heated Pd-catalyzed fluorination of aryl alcohols.

**Fluorination of arenes, aryl bromides, -alcohols, -triflates, and -boronic acid derivatives:** In 2013, Larhed and co-workers [51] established a one-pot, two-step fluorination of aryl alcohols via aryl nonafluorobutylsulfonates. This transformation employed Pd_2_(dba)_3_/*t*-BuBrettPhos and CsF to convert aryl alcohols to aryl fluorides at 180 °C under microwave conditions (Scheme 14). The proposed catalytic cycle of this aryl fluorination is also shown. Only reductive elimination was investigated by Larhed, because this reaction step is crucial for product formation and a successful outcome of the reaction.

**Scheme 15:**
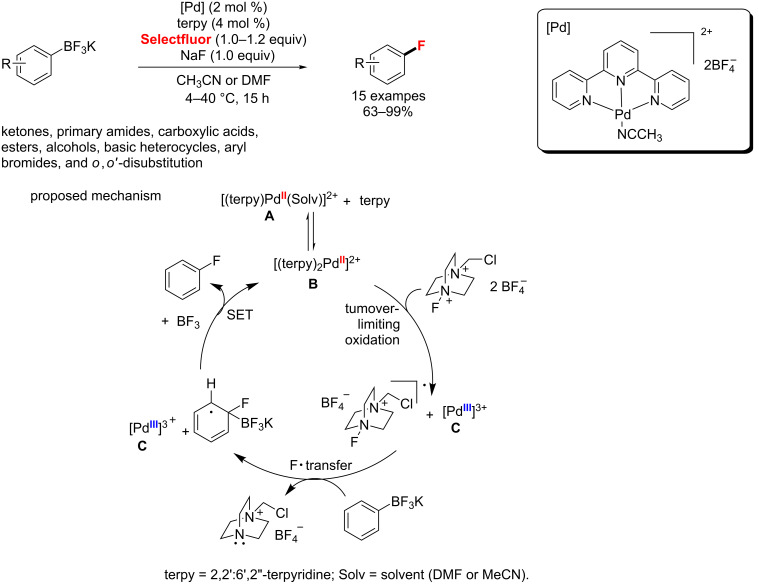
Fluorination of aryl potassium trifluoroborates.

In the same year, the Ritter group [52] reported a Pd-catalyzed fluorination of arylboronic acid derivatives via a Pd(II)/Pd(III) cycle (Scheme 15). A single-electron-transfer (SET) mechanism involving a well-defined Pd(III) intermediate has been proposed. First, a bis(terpyridyl)Pd(II) complex **B** is oxidized by Selectfluor with turnover-limiting to obtain Pd(III) **C** and a Selectfluor radical cation. Then, a transfer of a F^·^ radical from the Selectfluor radical cation to an aryl trifluoroborate occurs, forming the C−F bond and producing a delocalized radical. Finally, SET from the radical to **C** regenerates palladium species **B**, and affords a delocalized cation which converts to the aryl fluoride with loss of BF_3_. Notably, the addition of NaF increases the yield of aryl fluoride by reacting with the generated BF_3_.

**Scheme 16:**
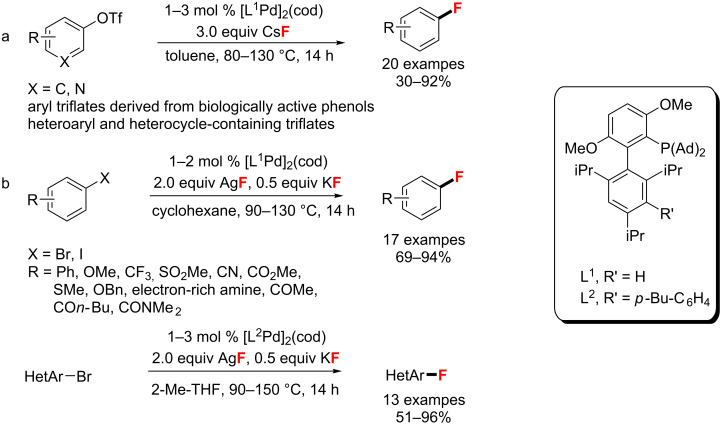
C(sp^2^)–F bond formation using precatalyst [L·Pd]_2_(cod).

In 2013, Buchwald et al. [53] introduced an improved catalyst system involving CsF and the stable Pd(0) species [(1,5-cyclooctadiene)(L^1^·Pd)_2_] (L^1^ = AdBrettPhos), which is a precatalyst for the fluorination of aryl triflates and heteroaryl triflates (Scheme 16a). Furthermore, aryl fluorides were provided in good to excellent yields with easy to separate byproducts. A year later, the same catalyst was employed for the nucleophilic fluorination of aryl bromides and iodides with AgF and KF [54]. Meanwhile, with a slight modification of the phosphine ligand, Buchwald developed a similar Pd(0) precatalyst [L^2^Pd]_2_(cod), which was used to fluorinate nitrogen-containing heteroaryl bromides (Scheme 16b).

**Scheme 17:**
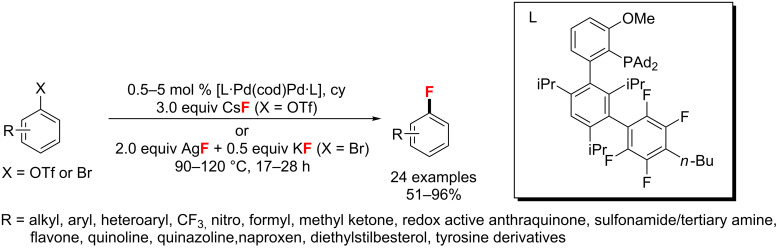
Pd-catalyzed fluorination of (hetero)aryl triflates and bromides.

In 2015, Buchwald and co-workers [55] explored a novel ligand for the Pd-catalyzed fluorination of (hetero)aryl triflates and bromides. The desired aryl fluorides were obtained with higher than 100:1 selectivity (Scheme 17).

**Scheme 18:**
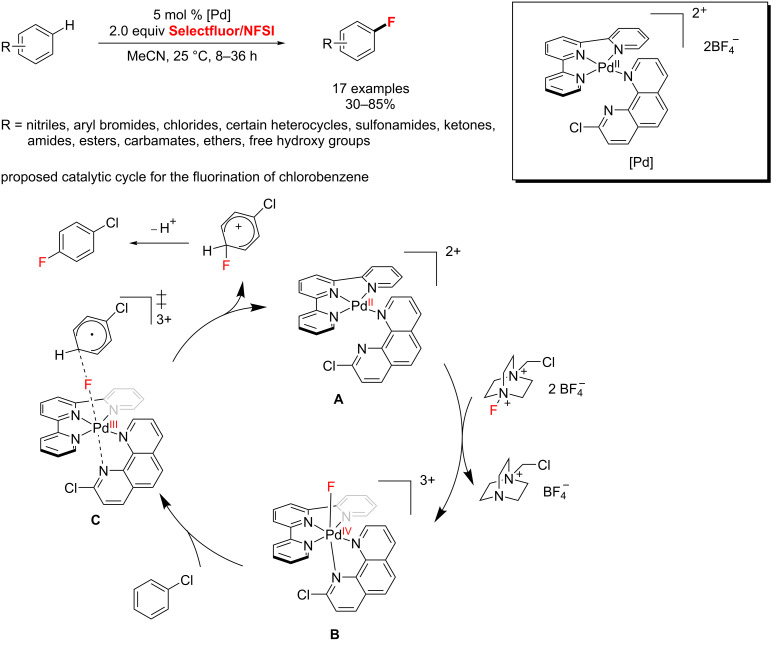
The Pd-catalyzed C–H fluorination of arenes with Selectfluor/NFSI.

More recently, Yamamoto and co-workers [56] described a palladium-catalyzed general method for aromatic C–H fluorination with mild electrophilic fluorinating reagents at room temperature (Scheme 18). Notably, in this process, a reactive transition metal fluoride electrophile **B** is catalytically formed from **A** with Selectfluor or NFSI instead of an organometallic intermediate as usual. Then, the activated Pd(IV)–F electrophile **B** would be capable of electrophilic fluorination of weakly nucleophilic arenes. This unusual mechanism of catalysis may provide a new idea to the catalysis of C–H functionalization reactions.

**Scheme 19:**
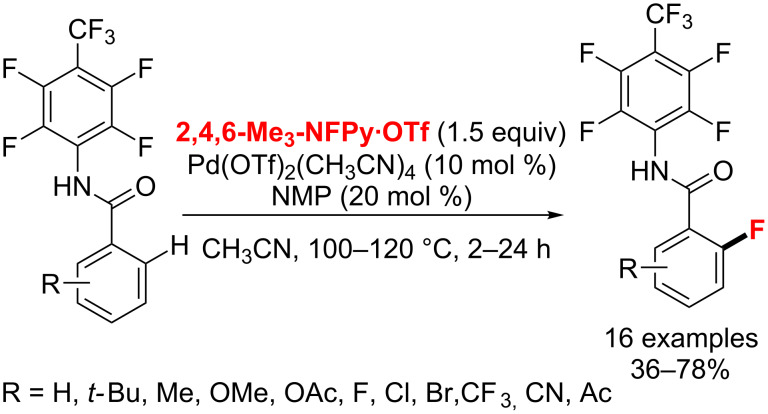
Pd(II)-catalyzed *ortho*-monofluorination protocol for benzoic acids.

**Aryl C–H fluorination with various directing groups:** With Pd(OTf)_2_(MeCN)_4_ and *N*-methyl-2-pyrrolidinone (NMP) used as the catalyst system, in 2011 the Yu group [57] described the *ortho*-fluorination of benzoic acid substrates with a directing group, an electron-deficient removable acidic amide (Scheme 19). With this method, both mono- and difluorinated benzoic acid derivatives can be selectively obtained in high yields.

**Scheme 20:**
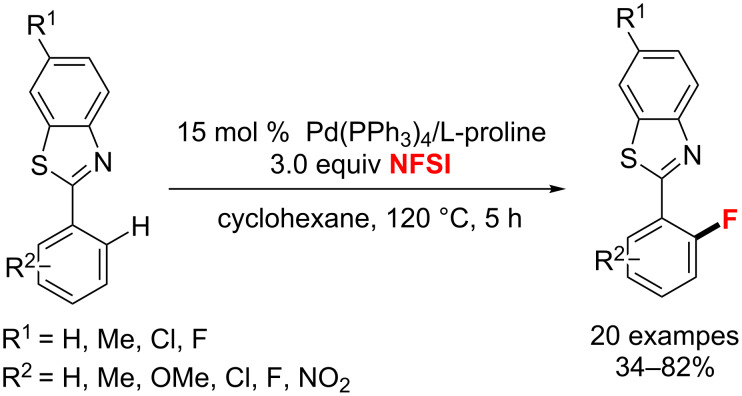
Pd-catalyzed C(sp^2^)–H bond fluorination of 2-arylbenzothiazoles.

In 2014, Pu and co-workers [58] devised the regioselective Pd(PPh_3_)_4_-catalyzed electrophilic *ortho*-fluorination of 2-arylbenzothiazoles with NFSI and ʟ-proline as the crucial promoter and the benzothiazoles as the directing groups (Scheme 20). This strategy plays an important role in the pharmaceutical and agrochemical industries.

**Scheme 21:**
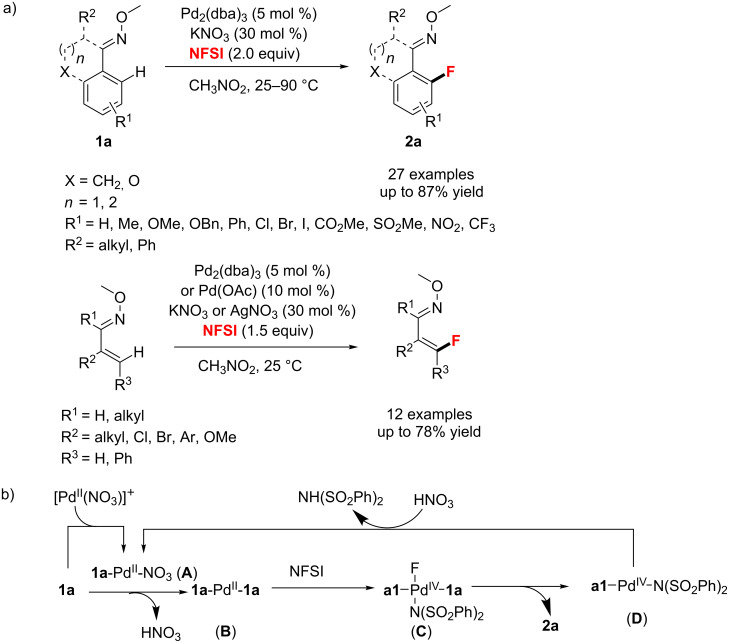
Nitrate-promoted fluorination of aromatic and olefinic C(sp^2^)–H bonds and proposed mechanism.

Meanwhile, Xu’s group [59] used *O*-methyl oxime as the directing group for the Pd-catalyzed *ortho*-fluorination of aromatic and olefinic C(sp^2^)–H bonds (Scheme 21a). It is worth noting that a cheap and nontoxic nitrate was added as a highly efficient promoter in the presence of NFSI and Pd_2_(dba)_3_. In addition, the authors proposed a reaction mechanism that involves a Pd(II)/Pd(IV) catalytic cycle (Scheme 21b). At the early stage of this process, an in situ-generated cationic [Pd(NO_3_)]^+^ species facilitates the C–H bond activation to give intermediate **A**. The Pd(II)(**1a**)_2_ complex **B** is formed via further C–H bond activation of another molecule **1a** by the cyclopalladation(II) intermediate **A**. Then, intermediate **B** undergoes oxidative addition by NFSI to give the highly reactive species F–Pd(IV)**1a**)_2_-N(SO_2_Ph)_2_ (**C**), which produces the product **2a** and reductive elimination intermediate **1a**-Pd(II)-N(SO_2_Ph)_2_ (**D**). Finally, intermediate **A** regenerates from intermediate **D** by aid of the catalytic amount of HNO_3_ released during the C–H activation step.

**Scheme 22:**
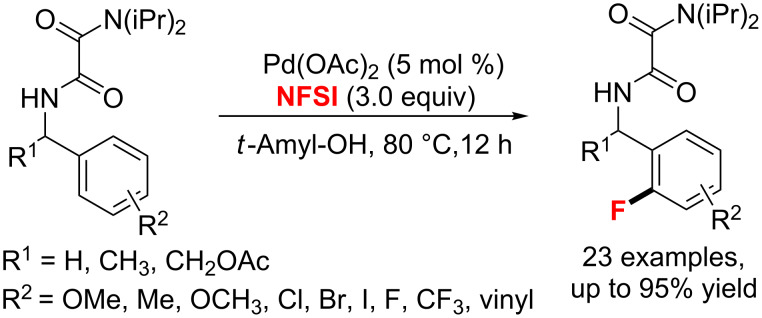
Fluorination of oxalyl amide-protected benzylamine derivatives.

In 2015, Zhao et al. [60] discovered a Pd(II)-catalyzed *ortho*-selective C–H fluorination of oxalyl amide-protected benzylamines (Scheme 22). The yields were up to 95% with NFSI as the [F^+^] source and *tert*-amyl alcohol as the solvent.

**Scheme 23:**
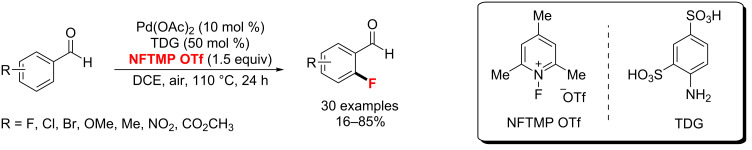
C–H fluorination of benzaldehydes with orthanilic acids as transient directing group.

In 2018, the Sorensen group [61] described a direct Pd-catalyzed *ortho*-C–H fluorination of benzaldehydes. Notably, these transformations were achieved with orthanilic acids as new transient directing groups (TDGs) in DCE in the presence of air (Scheme 23). This approach employed 1-fluoro-2,4,6-trimethylpyridinium salts as a bystanding F^+^ oxidant or an electrophilic fluorinating reagent. A broad substrate scope and high functional group compatibility were observed.

**Scheme 24:**
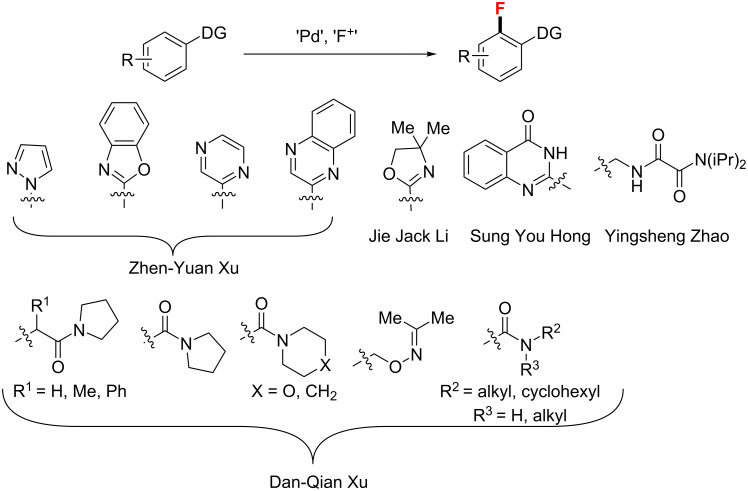
Pd(II)-catalyzed aryl C–H fluorination with various directing groups.

In addition to the methods discussed above, there are some other methods for the aromatic C–H fluorination using electrophilic fluorination reagents with various other directing groups [60,62–66]. Additionally, a diverse range of *N*-heterocycles, amides and motifs commonly encountered in medicinal chemistry were used as handles to direct C–H fluorination for the synthesis of pharmaceutical drugs (Scheme 24) [25].

#### Copper catalysis

Despite the success of Pd-catalyzed fluorinations, the more widespread use of these technologies has been partially hampered by the high cost and toxicity associated with Pd, in addition to the difficulty encountered when attempting to remove this metal from product mixtures. Therefore, cupper as low-cost, earth-abundant and readily available transition metal has emerged as a prevalent catalyst in a huge number of organic transformations. Similar to palladium complexes, copper complexes generally exist in four oxidation states, Cu(0), Cu(I), Cu(II), and Cu(III) and various fluorination reactions could be developed by different catalytic mechanisms.

**Scheme 25:**
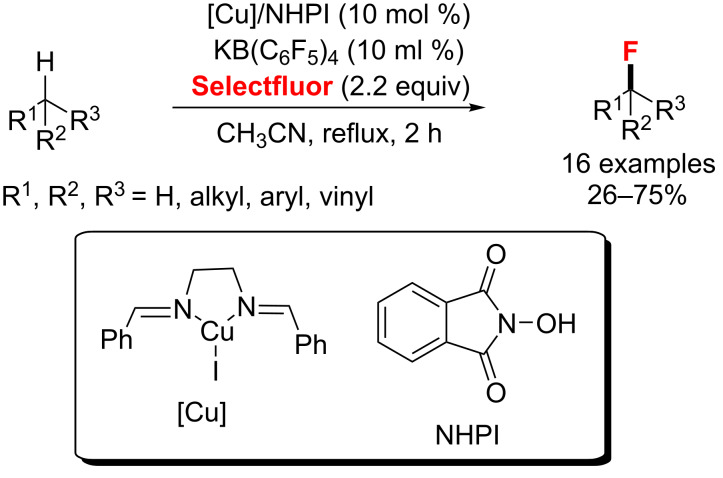
Cu-catalyzed aliphatic, allylic, and benzylic fluorination.

**Fluorination of inert C–H bonds, alkyl bromides and -triflates:** In a 2012 study, Lectka’s group [67] disclosed the catalytic fluorination of a series of aliphatic, benzylic, and allylic substrates with moderate yields. In this case, the authors employed a multicomponent catalytic system, involving Selectfluor, the radical precursor *N*-hydroxyphthalimide (NHPI), an anionic phase-transfer catalyst (KB(C_6_F_5_)_4_), and a Cu(I)-bisimine complex, to give the corresponding monofluorinated product (Scheme 25).

**Scheme 26:**
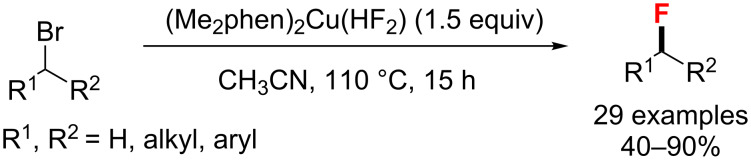
Cu-catalyzed S_N_2 fluorination of primary and secondary alkyl bromides.

One year later, Weng and co-workers [68] synthesized and characterized a new copper(I) fluoride complex ligated by a phenanthroline derivative. This complex was applied to the S_N_2 fluorination of primary and secondary alkyl bromides, producing the corresponding alkyl fluorides in 40–90% yield (Scheme 26).

**Scheme 27:**
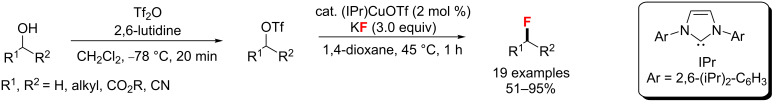
Copper-catalyzed fluorination of alkyl triflates.

In 2014, the group of Lalic [69] developed a mild fluorination of alkyl triflates with potassium fluoride catalyzed by a phase-transfer copper catalyst (Scheme 27). Notably, with 10 mol % of (IPr)CuOTf, full conversion can be accomplished in 10 minutes at 45 °C.

**Scheme 28:**
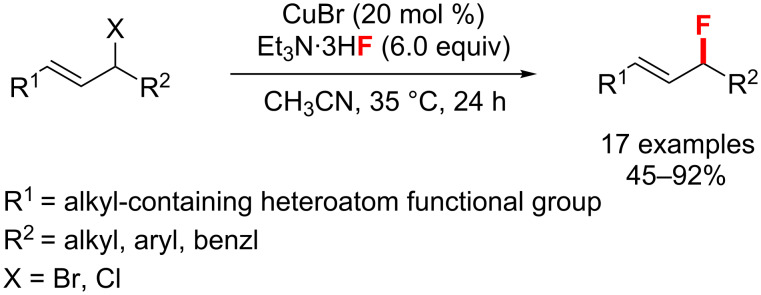
Cu-catalyzed fluorination of allylic bromides and chlorides.

**Allylic fluorination:** In 2013, there is an example of a copper-catalyzed fluorination of internal allylic bromides (Scheme 28). In Liu’s study, this approach was achieved using Et_3_N·3HF as the fluorine source with a high catalyst loading (20–30 mol %) affording the products in 45–92% yield [70]. The heteroatom-containing functional group (R^1^) is necessary for good reactivity and regioselectivity.

**Scheme 29:**
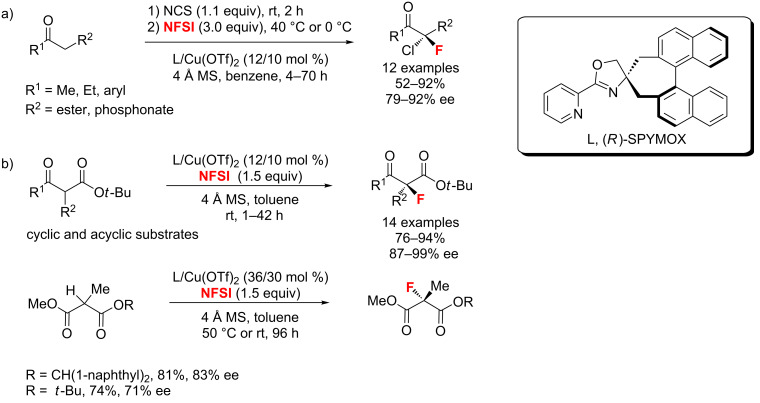
Synthetic strategy for the fluorination of active methylene compounds.

**α-Fluorination of acidic carbonyl compounds:** In 2011, Shibatomi and co-workers [71] described the one-pot asymmetric *gem*-chlorofluorination of active methylene compounds by using a copper(II) complex with a chiral spiro 2-pyridyl monooxazoline ligand (SPYMOX). The corresponding α-chloro-α-fluoro-β-keto esters were isolated with up to 92% ee (Scheme 29a). This approach could be extended to asymmetric *gem*-chlorofluorination of β-ketophosphonates. Two years later, the same authors [72] demonstrated the highly enantioselective fluorination of α-alkyl-β-keto esters and α-alkylmalonates using the same catalyst system (Scheme 29b). Moreover, various cyclic and acyclic substrates were successfully fluorinated with high enantioselectivities.

**Scheme 30:**
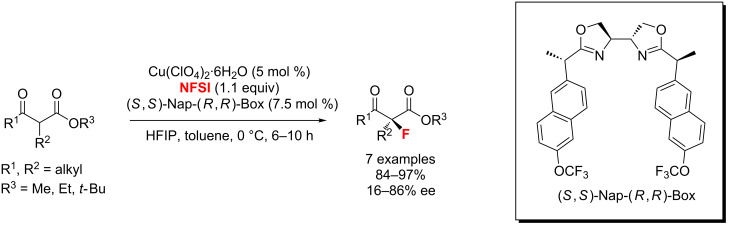
Fluorination of β-ketoesters using a tartrate-derived bidentate bisoxazoline-Cu(II) complex.

In 2013, the Kesavan group [73] reported the use of tartrate-derived bidentate bisoxazoline-Cu(II) complexes for the enantioselective fluorination of aliphatic cyclic and acyclic β-ketoesters with up to 98% yields (Scheme 30). In this method, (*S*,*S*)-Nap-(*R*,*R*)-Box as the most suitable diastereomeric ligand forms a 5-membered chelate with copper.

**Scheme 31:**
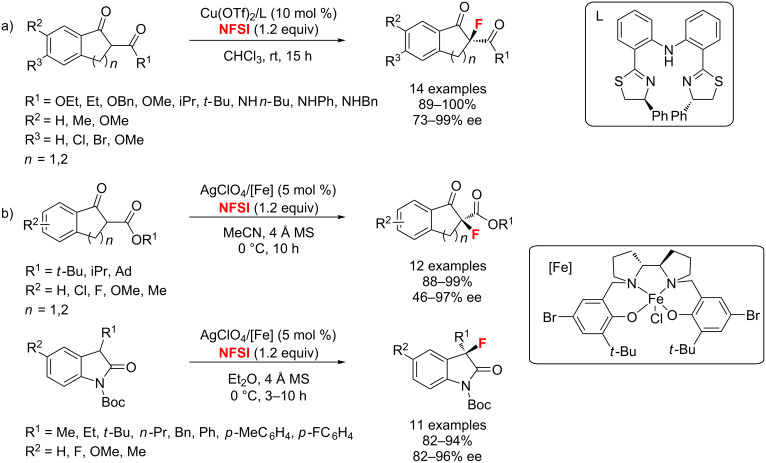
Highly enantioselective fluorination of β-ketoesters and *N*-Boc-oxindoles.

In the same year, an efficient and highly enantioselective fluorination of β-ketoesters catalyzed by diphenylamine-linked bis(thiazoline)-Cu(OTf)_2_ complexes was reported by Du and co-worker (Scheme 31a) [74]. Che and co-workers [75] achieved a similar α-fluorination of β-ketoesters and *N*-Boc-oxindoles (Scheme 31b). Compared with Du’s method, Che employed both AgClO_4_ and chiral iron(III)-salan complexes as the catalyst.

**Scheme 32:**
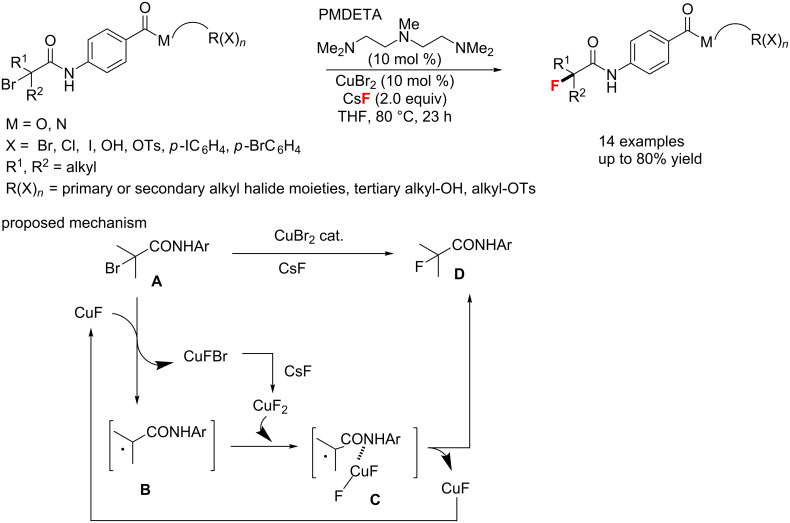
Amide group-assisted site-selective fluorination of α-bromocarbonyl compounds.

In 2016, the group of Nishikata [76] described a copper-catalyzed site-selective fluorination of α-bromocarbonyl compounds using a copper/CsF catalyst system (Scheme 32). Tertiary alkyl fluorides could be generated by this fluorination through the assistance of an amide group. From the results, the catalytic cycle of this reaction includes: 1) copper salt induced generation of the alkyl radical species **B** from substrate **A** and 2) fluorination of the alkyl radical species **B** with CuF_2_, which is in situ-generated from the reaction of CuXBr and CsF with the aid of an amide group, gives the desired product and recyclable CuF.

**Scheme 33:**
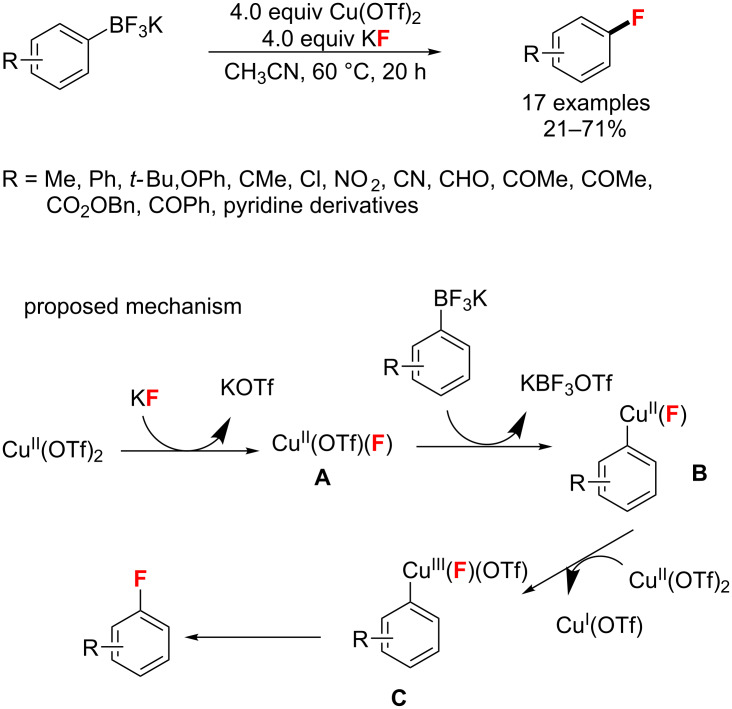
Cu-mediated aryl fluorination reported by Sanford [77].

**C_sp_^2^–H bond formation catalyzed by Cu catalysts:** In 2013, Sanford and co-workers [77] developed a simple and practical process for the nucleophilic fluorination of arylpotassium trifluoroborates. The reaction proceeds in CH_3_CN at 60 °C in the presence of Cu(OTf)_2_ as the catalyst and KF as the fluoride source (Scheme 33). A possible mechanism for this transformation is proposed in Scheme 33 below. Notably, Cu acts as both a mediator and an oxidizer in this reaction.

**Scheme 34:**
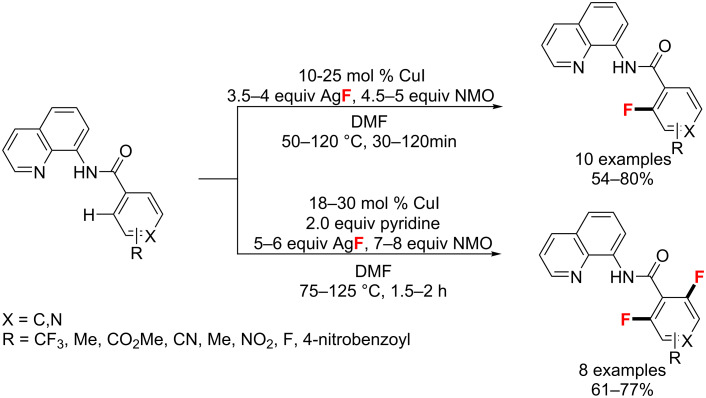
Mono- or difluorination reactions of benzoic acid derivatives.

In the same year, Daugulis et al. [78] presented a Cu-catalyzed selective fluorination of benzoic acid derivatives and benzylamine derivatives assisted by an aminoquinoline auxiliary. With a CuI catalyst, AgF as fluoride source, NMO as oxidant, and DMF as solvent, they achieved the selective mono- or difluorination in high yields (Scheme 34). Notably, pyridine as an additive could prevent the decomposition of an amide substrate in a long-time reaction.

**Scheme 35:**
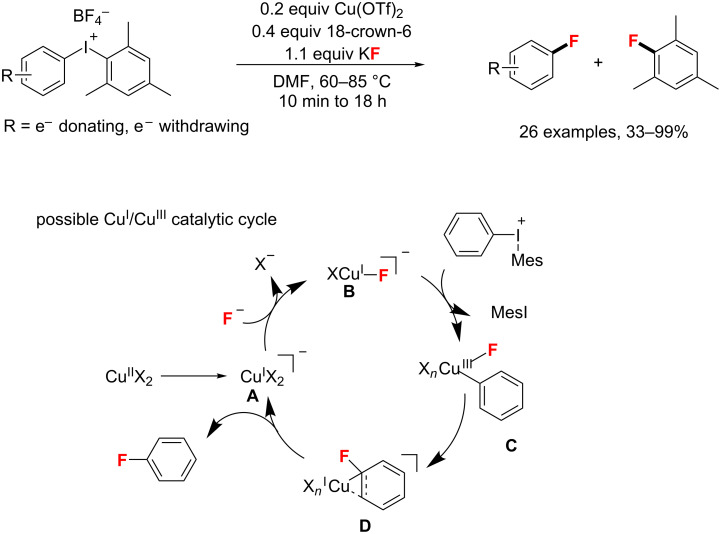
Cu-catalyzed fluorination of diaryliodonium salts with KF.

Meanwhile, the group of Sanford [79] presented the nucleophilic fluorination of diaryliodonium salts with KF through a Cu(I/III) catalytic cycle mechanism. This procedure preferentially fluorinates the smaller aromatic ligand on iodine(III). Also, the addition of Cu(OTf)_2_ and 18-crown-6 promoted the fluorination effectively. Finally, excellent yields, fast rate, high selectivity, and a broad substrate scope were observed by the authors (Scheme 35). The proposed mechanism is as follows: ligand exchange of the active Cu(I) catalyst **A**, which is generated via either reduction by the solvent or disproportionation of the precatalyst Cu(II)(OTf)_2_, provides Cu(I)-F (**B**). Then, oxidation of Cu(I)-F (**B**) by the diaryliodonium reagent forms Cu(III)–aryl intermediate **C**. Subsequently, a reductive elimination of intermediate **C** provides a putative π-complex **D**, which then releases the desired aryl–F product and regenerates the CuI catalyst **A**.

**Scheme 36:**
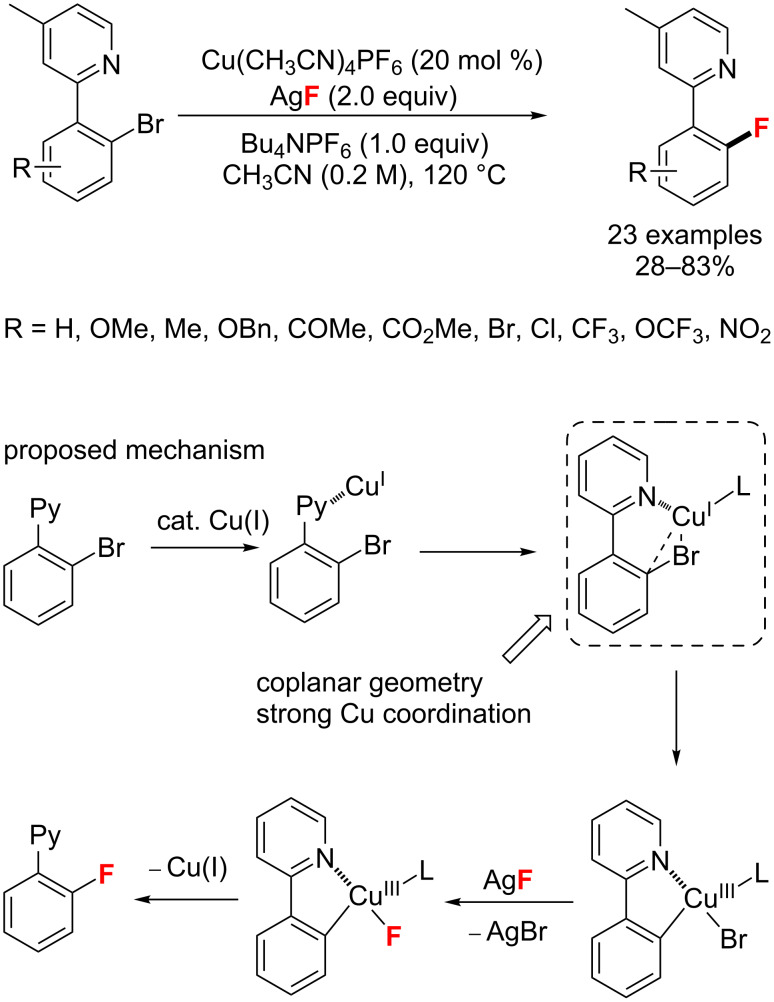
Copper(I)-catalyzed cross-coupling of 2-pyridylaryl bromides.

Subsequently, the Cu-catalyzed fluorination of 2-pyridylaryl bromides was achieved by Liu and co-workers [80] through a Cu(I/III) catalytic cycle as well (Scheme 36). This method is based on the aid of an important pyridyl directing group and the final aryl C–F bond is formed after the reductive elimination of ArCu(III)–F species.

#### Other catalysts

Other transition metals, including Co, Ni, Fe, Ag, Ir, Mn, etc., have received more and more attention.

**Scheme 37:**
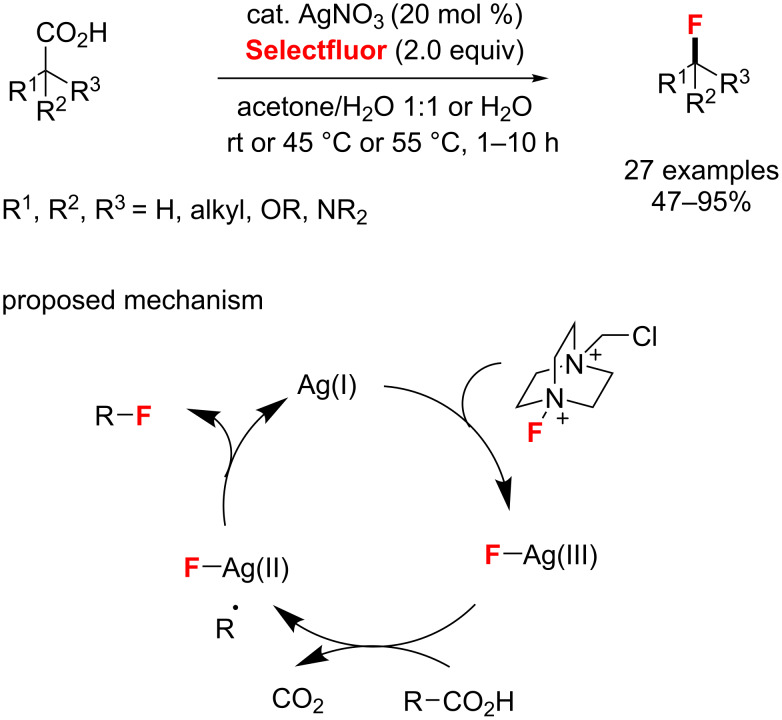
AgNO_3_-catalyzed decarboxylative fluorination of aliphatic carboxylic acids.

**Aliphatic and benzylic C–H fluorination and decarboxylative fluorination:** In 2012, a silver-catalyzed radical decarboxylative fluorination of aliphatic carboxylic acids in aqueous solution was provided by Li and co-workers (Scheme 37) [81]. The corresponding alkyl fluorides were produced in 47–95% yield under mild conditions. Additionally, the authors proposed a mechanism involving a Ag(III)-mediated SET followed by a fluorine transfer.

**Scheme 38:**
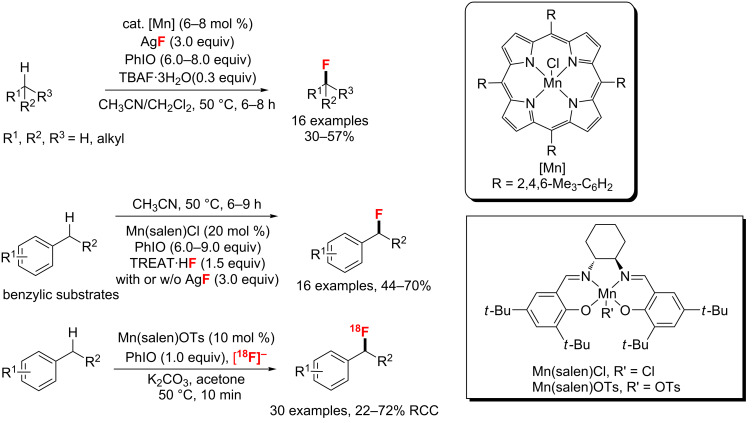
The Mn-catalyzed aliphatic and benzylic C–H fluorination.

Subsequently, the group of Groves [82] developed two manganese catalysts for the fluorination of C(sp^3^)–H bonds (Scheme 38). On the one hand, they employed a manganese porphyrin to catalyze the oxidative aliphatic C–H fluorination with iodosylbenzene (PhIO) as a stoichiometric oxidant. A variety of substrates, including simple hydrocarbons, substituted cyclic molecules, terpenoids, and steroid derivatives, were selectively fluorinated at some otherwise inaccessible sites, however, in low to moderate yields. On the other hand, the same group [83] developed Mn(salen)Cl as a catalyst for the direct C–H fluorination at benzylic positions with a nucleophilic fluorine source. Notably, Groves adapted the method for the ^18^F-radiofluorination of benzylic and aliphatic C–H bonds using no-carrier-added [^18^F]-fluoride with Mn(salen)OTs [84].

**Scheme 39:**
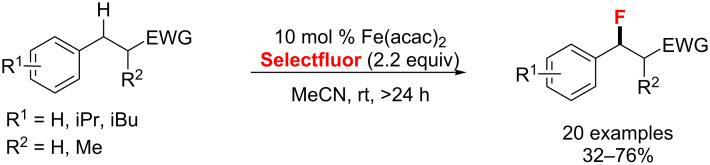
Iron(II)-promoted C–H fluorination of benzylic substrates.

In 2013, Lectka’s group [85–86] reported an iron-catalyzed C(sp^3^)–H fluorination of benzylic substrates with or without an electron-withdrawing group (EWG) in the presence of Selectfluor (Scheme 39). Notably, an EWG beta to the benzylic position is efficient for an excellent selectivity of the benzylic fluorination.

**Scheme 40:**
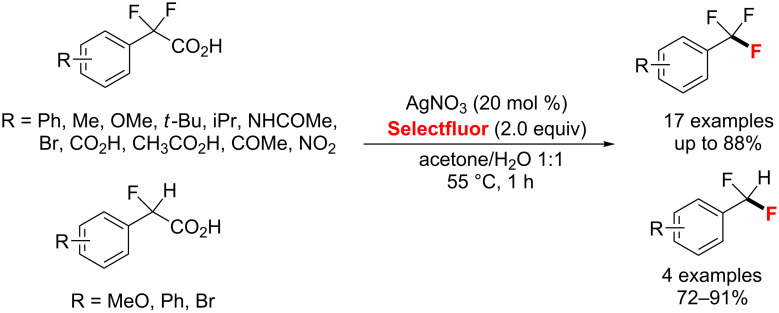
Ag-catalyzed fluorodecarboxylation of carboxylic acids.

Moreover, Gouverneur and co-workers [87] established the decarboxylative fluorination of α,α-difluoro- and α-fluoroarylacetic acids with a wide functional group compatibility in the presence of AgNO_3_ as catalyst in good yields (Scheme 40). Further, this approach was efficiently applied to the preparation of [^18^F]-labelled tri- and difluoromethylarenes using [^18^F]Selectfluor bis(triflate).

**Scheme 41:**
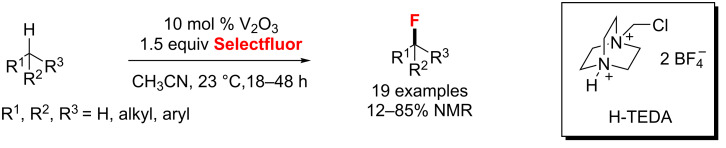
Vanadium-catalyzed C(sp^3^)–H fluorination.

In 2014, Chen and co-workers [88] described a selective direct C(sp^3^)–H fluorination catalyzed by a commercially available vanadium(III) oxide with Selectfluor in good yields (Scheme 41). It is noteworthy that the catalyst and the byproduct H-TEDA could be removed easily by filtration.

**Scheme 42:**
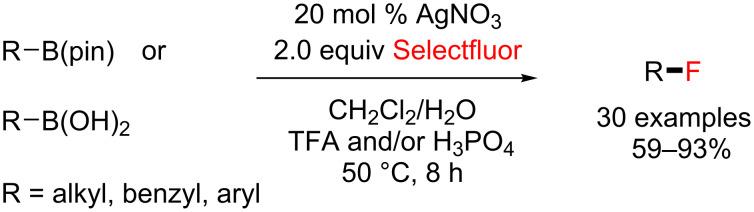
AgNO_3_-catalyzed radical deboronofluorination of alkylboronates and boronic acids.

A simple AgNO_3_-catalyzed synthesis of alkyl fluorides through radical deboronofluorination of alkyl boronates and boronic acids in acidic aqueous solution was also developed by Li and co-workers in 2014 [89]. This method features good yields and a wide functional group compatibility (Scheme 42).

**Scheme 43:**
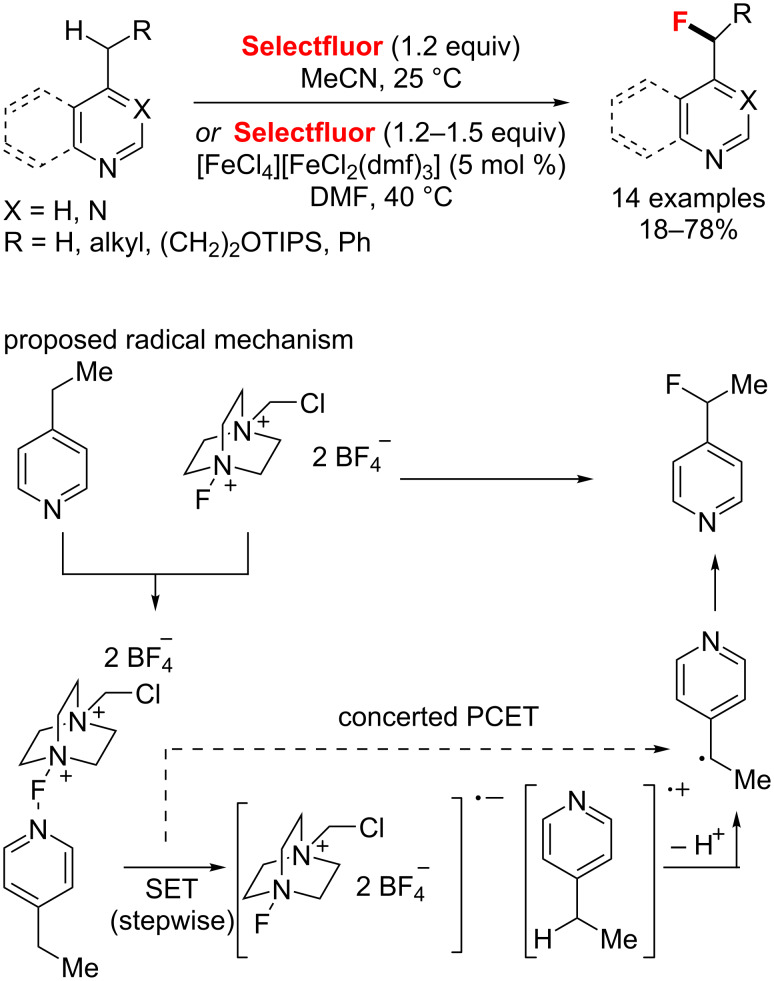
Selective heterobenzylic C–H fluorination with Selectfluor reported by Van Humbeck.

Recently, the group of Van Humbeck [90] reported a selective and mild method for the C–H fluorination of azaheterocycles with Selectfluor at room temperature. In this case, a new radical mechanism was proposed that electron transfer from the heterocyclic substrate to Selectfluor eventually generates a benzylic radical, leading to the desired C–F bond formation. The excellent selectivity of the desired fluorinated product was obtained without additives. In addition, a catalytic amount of iron(III) complex [FeCl_4_][FeCl_2_(dmf)_3_] was found to improve the yields in some cases (Scheme 43).

**Scheme 44:**
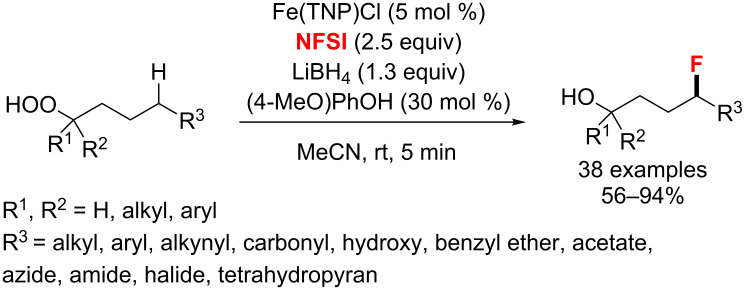
Fe(II)-catalyzed site-selective fluorination guided by an alkoxyl radical.

With an Fe(II)-catalyzed orchestrated redox process, an alkoxyl radical-guided strategy for the site-selective fluorination of unactivated methylene and methine C–H bonds was published by Liu and co-workers in 2018 (Scheme 44) [91]. The fluorination of various primary, secondary, and tertiary hydroperoxides was achieved in moderate to excellent yields, with the hydroperoxide functional group acting as a precursor of an alkoxy radical to control site-selective carbon-centered radical formation.

**Scheme 45:**
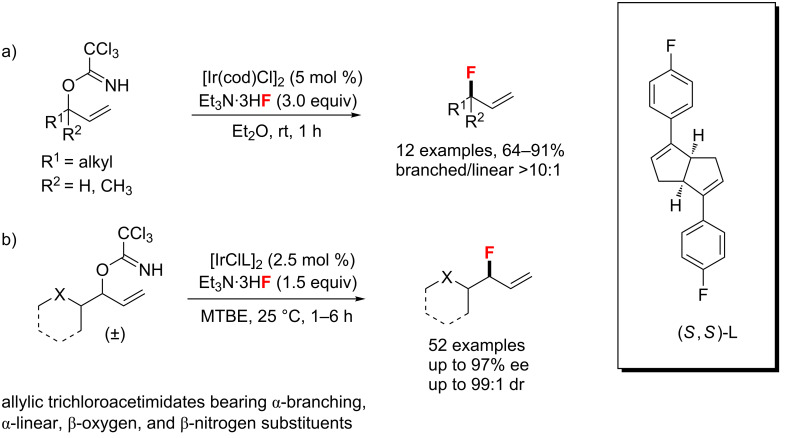
Fluorination of allylic trichloroacetimidates reported by Nguyen et al.

**Allylic fluorination:** In 2011, the group of Nguyen [92] developed the nucleophilic fluorination of allylic trichloroacetimidates, as shown in Scheme 45a. Cyclooctadiene iridium chloride dimer, [IrCl(COD)]_2_, was an effective catalyst to promote this fluorination with Et_3_N·3HF, forming allylic fluorides in moderate to good yields. This facile method shows a good regioselectivity to gain the branched isomer within 1 h. Later in 2017, they described a similar method for the asymmetric fluorination of racemic allylic trichloroacetimidates utilizing a chiral bicyclo[3.3.0]octadiene-ligated iridium complex (Scheme 45b) [93]. This reaction proceeded under mild conditions with an extremely broad substrate scope, as well as excellent branched-to-linear ratios and enantioselectivities.

**Scheme 46:**
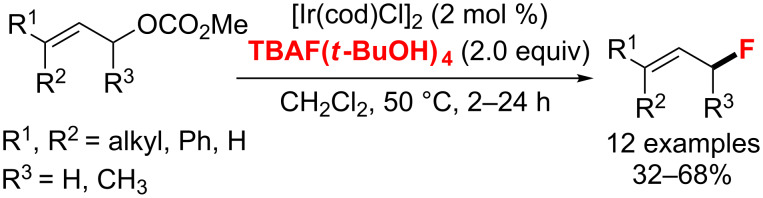
Iridium-catalyzed fluorination of allylic carbonates with TBAF(*t*-BuOH)_4_.

In 2013, Gouverneur and co-workers [94] demonstrated the regio and stereocontrolled fluorination of allylic carbonates with [Ir(COD)Cl]_2_ as the catalyst and TBAF(*t*-BuOH)_4_ as the fluoride source to produce branched and linear allylic fluorides (Scheme 46). Remarkably, this was the first example to afford (*Z*)-allyl fluorides (*Z*:*E* ratio > 20:1).

**Scheme 47:**
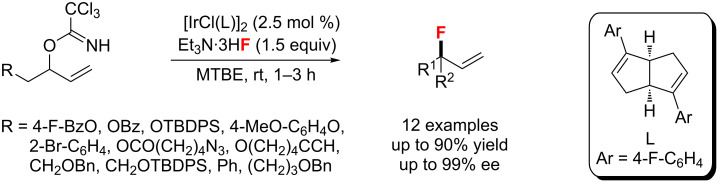
Iridium-catalyzed asymmetric fluorination of allylic trichloroacetimidates.

In 2015, Nguyen et al. [95] explored the asymmetric fluorination of racemic, secondary allylic trichloroacetimidates with Et_3_N·3HF using a chiral-diene-ligated Ir complex (Scheme 47). This process proceeded under mild conditions with excellent enantioselectivity and yields, a broad substrate scope, as well as a wide range of functional group compatibility. Notably, this strategy overcomes the challenges associated with the formation of secondary allylic fluorides bearing α-linear substituents, providing complete regio and stereocontrolled acrylic allylic fluorides.

**Scheme 48:**
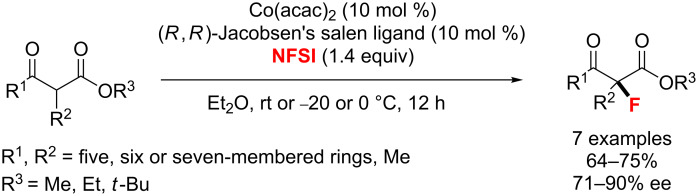
Cobalt-catalyzed α-fluorination of β-ketoesters.

**Fluorination of acidic carbonyl compounds:** In 2010, Itoh and co-workers [96] demonstrated the asymmetric fluorination of cyclic and acyclic β-ketoesters by using a catalytic amount of Co(acac)_2_ with (*R*,*R*)-Jacobsen’s salen ligand (Scheme 48). The α-fluorinated products were thus obtained with good enantioselectivity.

**Scheme 49:**
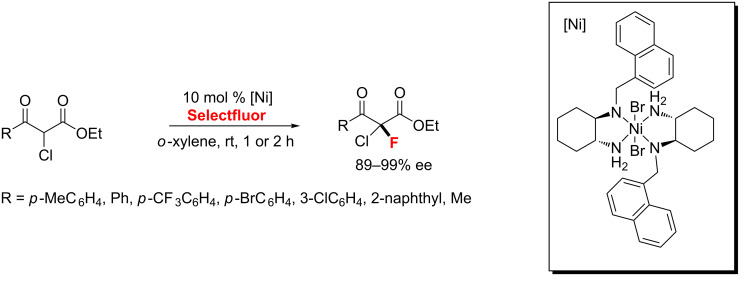
Nickel-catalyzed α-fluorination of various α-chloro-β-ketoesters.

In the same year, Kim’s group [97] accomplished an efficient enantioselective electrophilic α-fluorination of various α-chloro-β-ketoesters catalyzed by chiral nickel complexes with good enantioselectivity (up to 99% ee). Notably, the chiral nickel-diamine complexes are air and moisture-stable (Scheme 49).

**Scheme 50:**
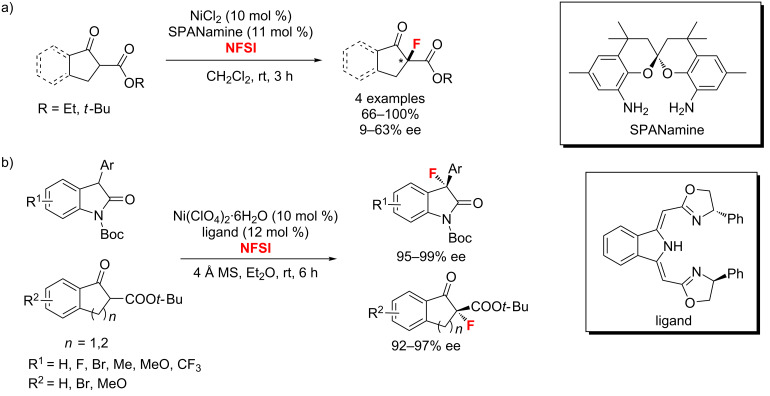
Ni(II)-catalyzed enantioselective fluorination of oxindoles and β-ketoesters.

In 2011, two nickel-catalyzed protocols for the enantioselective α-fluorination of β-ketoesters were reported separately. In van Leeuwen’s reaction, SPANamine derivatives were synthesized and applied as chiral ligands in the asymmetric α-fluorination of β-ketoesters (Scheme 50a) [98]. Meanwhile, to achieve this transformation, Gade and co-workers [99] developed a new class of chiral tridentate N-donor pincer ligands, bis(oxazolinylmethylidene)isoindolines. They obtained the desired products under mild conditions with excellent enantioselectivities (up to >99% ee) and good yields (Scheme 50b).

**Scheme 51:**
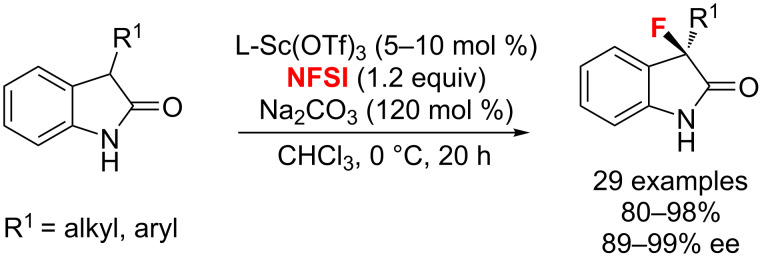
Scandium(III)-catalyzed asymmetric C–H fluorination of unprotected 3-substituted oxindoles.

Also, Feng et al. [100] developed a new method for the highly enantioselective fluorination of N–H-free 3-substituted oxindoles catalyzed by a Sc(III)/*N*,*N*’-dioxide complex. A series of 3-aryl- and 3-alkyl-3-fluoro-2-oxindoles were obtained in excellent yields and enantioselectivities (89–99% ee) with NFSI under basic conditions (Scheme 51).

**Scheme 52:**
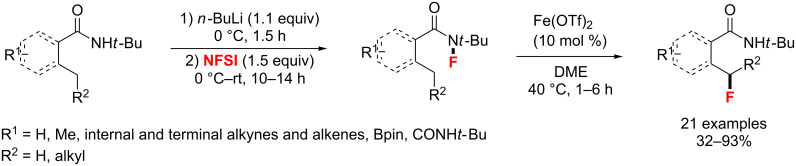
Iron-catalyzed directed C–H fluorination.

In 2016, a mild, amide-directed fluorination of benzylic, allylic, and unactivated C–H bonds was described by the Cook group [101]. By the use of the iron(II) triflate (Fe(OTf)_2_) as catalyst, the desired fluorides were finally obtained through a F-transfer of a short-lived radical intermediate (*N*-fluoro-2-methylbenzamides) in up to 93% yield (Scheme 52).

**Scheme 53:**
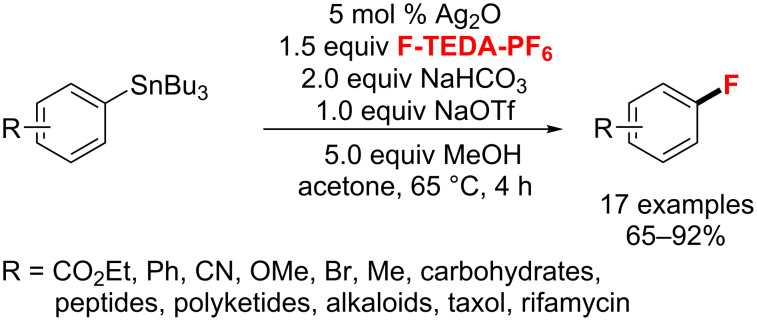
Electrophilic silver-catalyzed Ar–F bond-forming reaction from arylstannanes.

**C_sp_^2^–H bond formation catalyzed by Ag catalysts:** In 2010, the Ritter group [102] firstly reported a Ag-catalyzed fluorination of arylstannane derivatives with the electrophilic fluorination reagent F-TEDA-PF_6_ (Scheme 53). Also, the reaction was applied to late-stage fluorination of small molecules. However, this method uses toxic arylstannanes as starting materials and requires an additional synthetic step from the triflate or halide to the stannanes.

### Trifluoromethylation

Transition-metal-catalyzed trifluoromethylation reactions have made great progress in the joint efforts of organic fluorination scientists and metalorganic chemists over the past decade. Introducing trifluoromethyl groups into organic molecules can significantly alter their properties, such as their metabolic stability, lipophilicity, and the ability to penetrate the blood–brain barrier. Similar to fluorination, trifluoromethylation can also be achieved by three reaction types: nucleophilic, electrophilic and radical trifluoromethylation.

**Figure 1:**
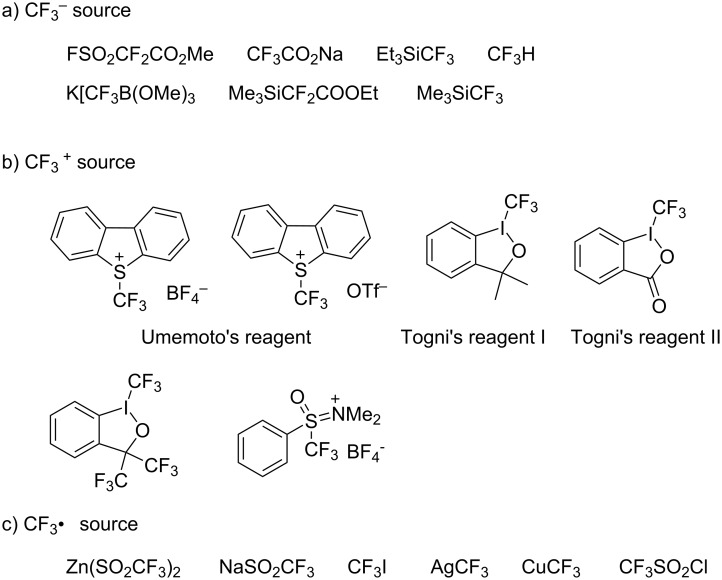
Nucleophilic, electrophilic and radical CF_3_ sources.

In recent years, many novel trifluoromethylation reagents, such as cationic, anionic and radical CF_3_ sources have been discovered and offer manifold choices to effect electrophilic, nucleophilic and radical trifluoromethylation [103] (Figure 1). The selection of the trifluoromethylation reagent has become the main factor in the optimization of these reactions. With a suitable trifluoromethylation reagent, a wide range of substrates are directly converted to the desired trifluoromethylated products. Several reviews [104–110] have been published on this subject, while this part mainly discusses trifluoromethylation reactions catalyzed by metals. However, there are only a few methods available for the C(sp^3^)–CF_3_ bond formation and this transformation still needs further examination.

#### C(sp^3^)–CF_3_ bond formation

**Scheme 54:**
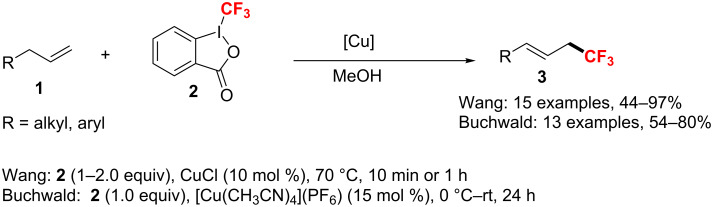
Cu(I)-catalyzed allylic trifluoromethylation of unactivated terminal olefins.

**Copper catalysis:** In 2011, two Cu(I)-catalyzed allylic trifluoromethylation reactions of terminal olefins have been developed independently by the groups of Buchwald [111] and Wang [112] (Scheme 54). Under similar mild conditions using Togni's reagent II, the desired allyl–CF_3_ products were obtained and the methods well tolerated a variety of functional groups (e.g., esters, epoxides, amides, alcohols, or aldehydes). Moreover, the thermodynamically favored *E*-olefin was generated with high stereoselectivity in good yields.

**Scheme 55:**
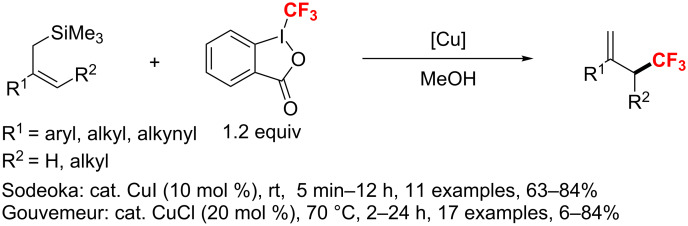
Direct copper-catalyzed trifluoromethylation of allylsilanes.

In 2012, two different groups [113–114] individually reported the direct trifluoromethylation of allylsilanes under very similar conditions. These processes furnished various branched cyclic and acyclic allylic CF_3_ products using copper as the catalyst (Scheme 55).

**Scheme 56:**
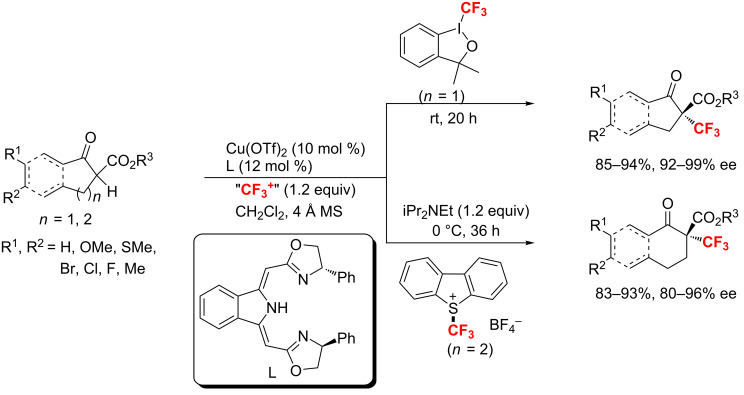
Cupper-catalyzed enantioselective trifluoromethylation of five and six-membered ring β-ketoesters.

Subsequently, an enantioselective trifluoromethylation of cyclic β-ketoesters with commercially available trifluoromethylating reagents was reported by Gade and co-workers using a Cu-boxmi catalyst [115]. Under mild conditions, both five and six-membered ring β-ketoesters were converted to the corresponding products in high yields and enantioselectivities (Scheme 56).

**Scheme 57:**
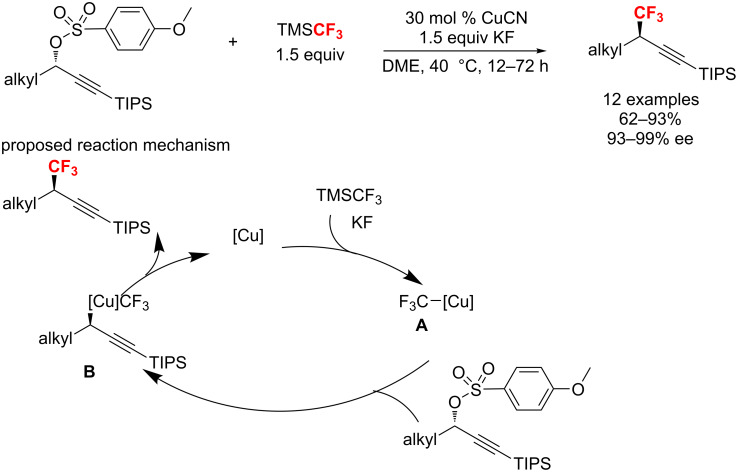
Cu-catalyzed highly stereoselective trifluoromethylation of secondary propargyl sulfonates.

In 2018, the first example for the copper-catalyzed stereospecific trifluoromethylation of secondary propargyl sulfonates was described by the group of Zhang [116]. The resulting chiral trifluoromethylated alkynes were acquired with high regioselectivity and stereospecificity (ees up to >99%). Furthermore, this reaction showed a broad substrate scope, as well as excellent functional-group compatibility (Scheme 57). A possible mechanism was proposed: firstly, trifluoromethylcopper complex A, generated from CuCN with TMSCF_3_, undergoes oxidative addition with a secondary propargyl sulfonate to give a configuration-inversed propargyl-Cu(III) species B. Then, the reductive elimination of B affords the final product with overall inversion of the configuration.

**Scheme 58:**
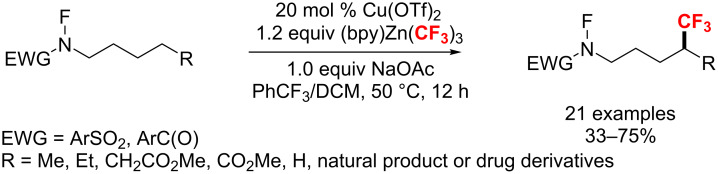
Remote C(sp^3^)–H trifluoromethylation of carboxamides and sulfonamides.

Recently, Li and co-workers [117] explored a simple and facile method to access δ-trifluoromethylated carboxamides and sulfonamides through a copper-catalyzed 1,5-hydrogen atom transfer (Scheme 58).

**Scheme 59:**
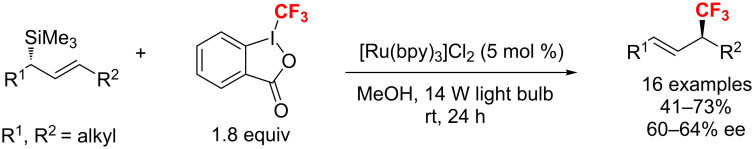
Trifluoromethylation of allylsilanes with photoredox catalysis.

**Other catalysts:** In 2013, Gouverneur and co-workers [118] described a photoredox-based catalytic approach to afford enantioenriched branched allylic CF_3_ products from allylsilanes using [Ru(bpy)_3_]Cl_2_ (Scheme 59). Herein, the silyl group in the substrate plays an important role in controlling the regioselectivity of the trifluoromethylation reaction.

**Scheme 60:**
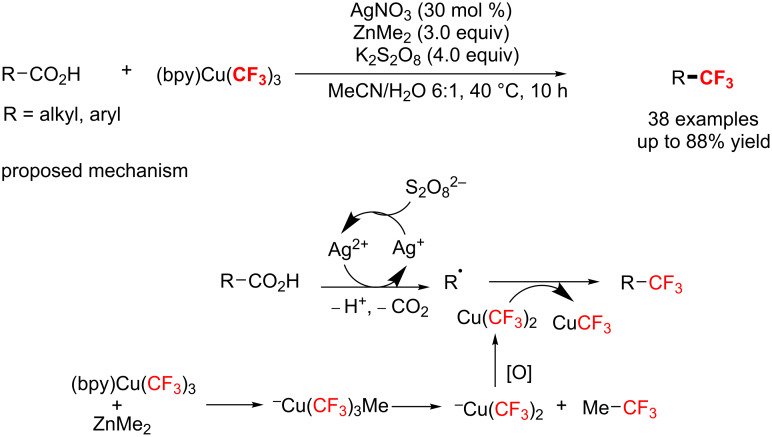
Ag-catalyzed decarboxylative trifluoromethylation of aliphatic carboxylic acids in aqueous CH_3_CN.

Later in 2017, Li’s group [119] described a practical protocol for the decarboxylative trifluoromethylation of various primary and secondary aliphatic carboxylic acids. With AgNO_3_ as a catalyst, (bpy)Cu(CF_3_)_3_ (bpy = 2,2’-bipyridine) as a CF_3_ source and K_2_S_2_O_8_ as an oxidant, aliphatic carboxylic acids were converted to the corresponding trifluoromethylated products in good yields (Scheme 60). Also, mechanistic studies, a radical clock experiment, revealed the intermediacy of ^−^Cu(CF_3_)_3_Me, which undergoes reductive elimination and subsequent oxidation to give the active species Cu(CF_3_)_2._ Meanwhile, aliphatic carboxylic acids give the corresponding alkyl radicals via Ag(II)-mediated oxidative decarboxylation. Then, Cu(CF_3_)_2_ provides a CF_3_ group to alkyl radicals to obtain the final product.

**Scheme 61:**
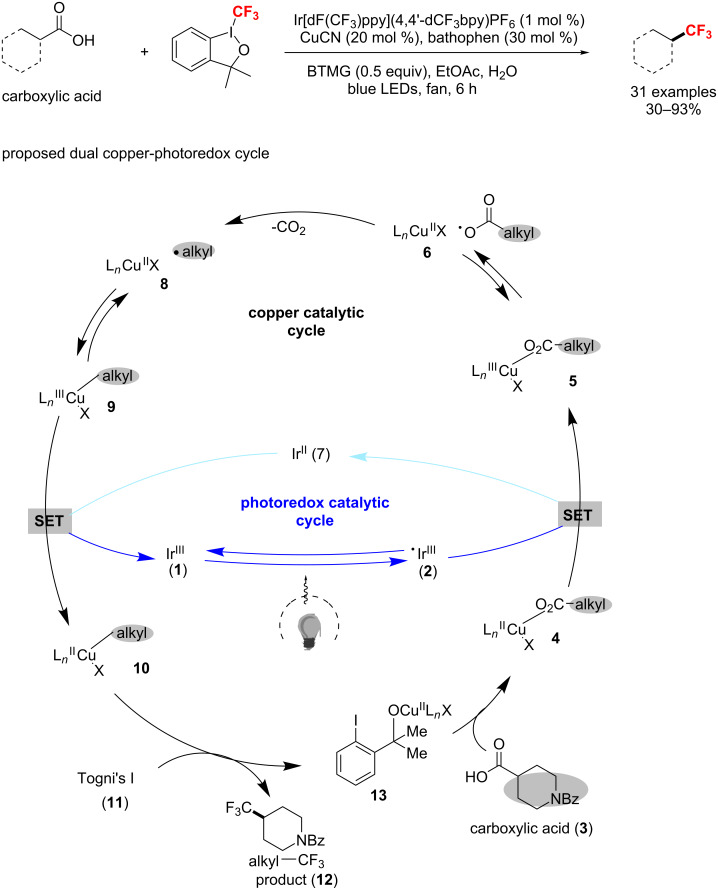
Decarboxylative trifluoromethylation of aliphatic carboxylic acids via combined photoredox and copper catalysis.

Very recently, MacMillan et al. [120] discovered an efficient approach to the decarboxylative trifluoromethylation of aliphatic carboxylic acids via the combination of photoredox and copper catalysis (Scheme 61). The method tolerates a myriad of primary, secondary and tertiary carboxylic acids and provides the corresponding CF_3_ analogue in good to excellent yields. Details of the proposed dual copper–photoredox cycle are shown in Scheme 61. The Ir(III) photocatalyst Ir[dF(CF_3_)ppy]_2_(4,4’-dCF_3_bpy)PF_6_ (**1**) undergoes photoexcitation with visible light to form the highly oxidizing excited state **^·^**Ir(III) **2**. Then, SET from copper carboxylate **4**, derived from carboxylic acid **3** with the Cu(II) catalyst to **^·^**Ir(III) **2** provides Cu(III) carboxylate **5**, or in the dissociated form, a carboxyl radical and Cu(II) complex **6**, along with reduced Ir(II) photocatalyst **7**. The resulting carboxyl radical extrudes CO_2_ and sequentially recombines to generate Cu(III) species **9**. At this stage, SET from **7** to **9** closes the photoredox catalytic cycle and produces an alkylcopper(II) species **10**. Under the addition of Togni’s reagent I (**11**), species **10** affords the final alkyl−CF_3_ product and complex **13**, which is used for ligand exchange with **3**.

#### C(sp^2^)–CF_3_ bond formation

**Scheme 62:**
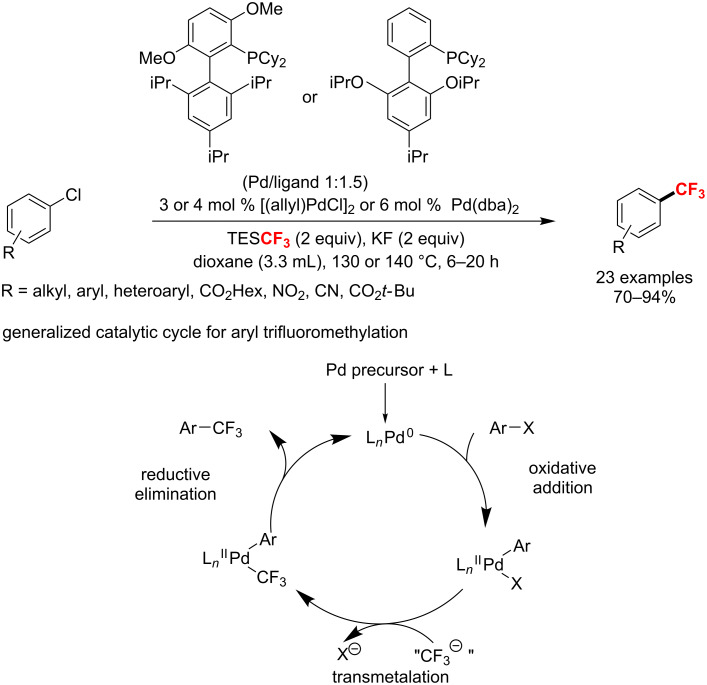
Palladium-catalyzed Ar–CF_3_ bond-forming reaction.

**Palladium-catalyzed trifluoromethylation of aryl and vinyl compounds:** In 2010, Watson and co-workers [121] developed the first Pd-catalyzed trifluoromethylation of aryl/heterocyclic chlorides with the CF_3_ source TESCF_3_ (TES, triethylsilyl), which proceeded following a classical Pd(0)/Pd(II) catalytic cycle (Scheme 62). Also, the reaction tolerates a variety of functional groups, such as esters, amides, ethers, nitriles, etc., and therefore provides a new way for late-stage modifications.

**Scheme 63:**
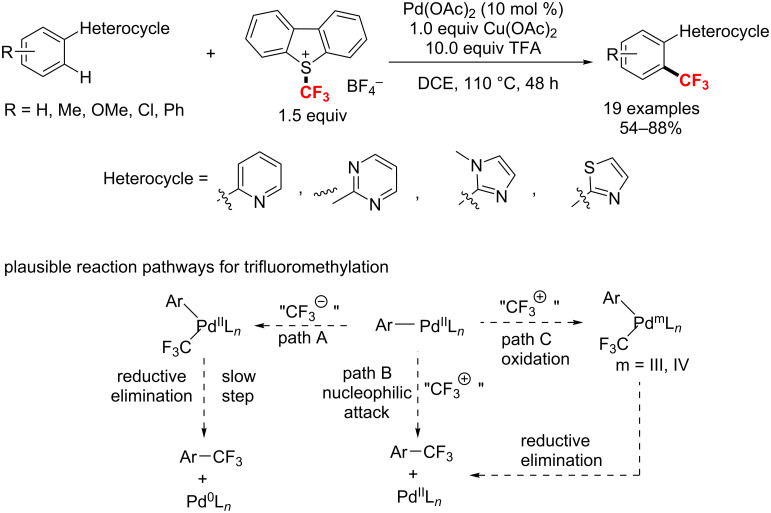
Palladium-catalyzed trifluoromethylation of arenes with diverse heterocyclic directing groups.

In the same year, Yu’s group [122] reported a Pd(II)-catalyzed C–H trifluoromethylation of arenes with an electrophilic trifluoromethylation reagent using diverse heterocyclic directing groups. Notably, the presence of trifluoroacetic acid (TFA) is crucial for the Ar–CF_3_ bond formation and Cu(OAc)_2_ can increase the catalytic turnover (Scheme 63). Based on three different modes of the ArPd(II) species reaction with nucleophiles, electrophiles and highly oxidizing reagents, three possible reaction pathways (A, B and C, respectively) are envisaged, that can follow the C–H activation event to the trifluoromethylated products, as described in Scheme 63. In this case, the specific catalytic mechanism remains to be studied.

**Scheme 64:**
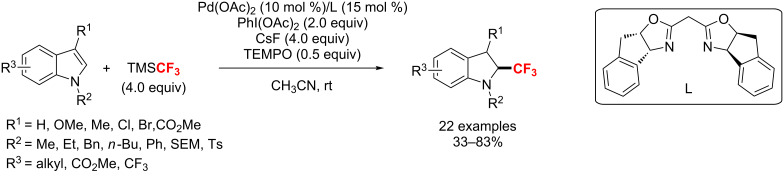
Pd-catalyzed trifluoromethylation of indoles as reported by Liu.

In 2011, the group of Liu [123] accomplished a Pd(II)-catalyzed oxidative trifluoromethylation of indoles with TMSCF_3_ and PhI(OAc)_2_ at room temperature (Scheme 64). Through reductive elimination from the (Ar)Pd(IV)-CF_3_ intermediate, the aryl C–CF_3_ bond is generated. Notably, the bidentate nitrogen-containing ligand is crucial to the achievement of this process.

**Scheme 65:**
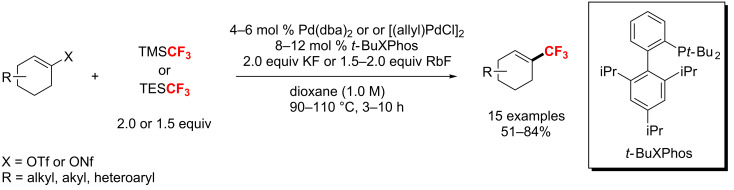
Pd-catalyzed trifluoromethylation of vinyl triflates and vinyl nonaflates.

In the same year, Buchwald et al. [124] discovered a palladium-catalyzed trifluoromethylation of vinyl triflates and nonaflates (Scheme 65). A variety of trifluoromethylated cyclohexenes were obtained using a catalyst system, which was composed of Pd(dba)_2_ or [(allyl)PdCl]_2_ and the monodentate biaryl phosphine ligand *t*-BuXPhos. Also, TMSCF_3_ and KF were more suitable to the trifluoromethylation of triflate electrophiles, while the use of TESCF_3_ and RbF gave better results for nonaflate electrophiles.

**Scheme 66:**
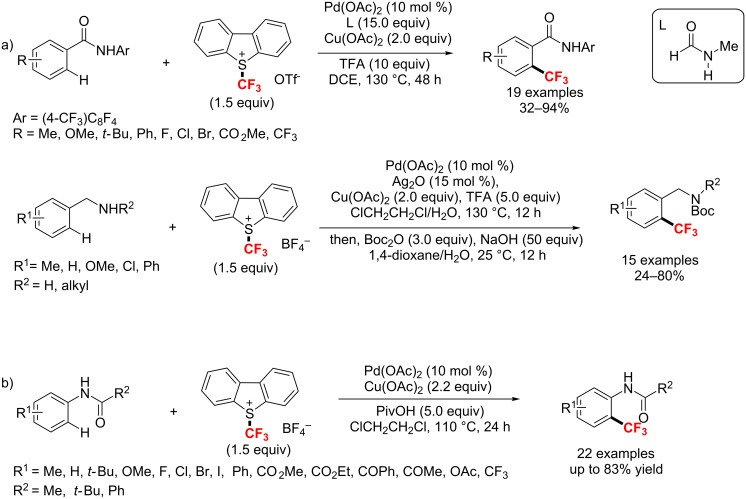
Pd(II)-catalyzed *ortho*-trifluoromethylation of aromatic C–H bonds.

Subsequently, the Yu [15,125] and Shi group [126] independently reported the palladium-catalyzed *ortho*-trifluoromethylation of an aromatic C–H bond with Umemoto’s trifluoromethylation reagent. Notably, Cu(II) salts were crucial for forming the aryl–CF_3_ bonds. In Yu’s study, benzamides and benzylamines were well trifluoromethylated via a Pd(II)/Pd(IV) catalytic cycle with the addition of TFA (and Ag_2_O) (Scheme 66a). With an acetamido group as a directing group, Shi developed an efficient method to access *ortho*-CF_3_ acetanilides and anilines (Scheme 66b).

**Scheme 67:**
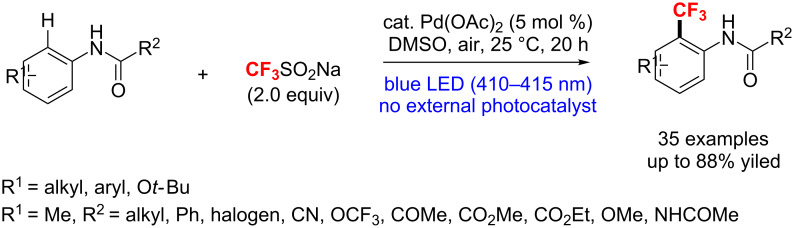
Visible-light-induced Pd(OAc)_2_-catalyzed *ortho*-trifluoromethylation of acetanilides with CF_3_SO_2_Na.

Recently, Wang and co-workers [127] reported a visible-light-induced Pd-catalyzed *ortho*-trifluoromethylation of acetanilides. Without the need of an external photocatalyst and additive, various N-substituted anilides and acetanilides were obtained efficiently at room temperature in air. The strategy features good yields, broad functional group tolerance and high regioselectivity (Scheme 67).

**Scheme 68:**
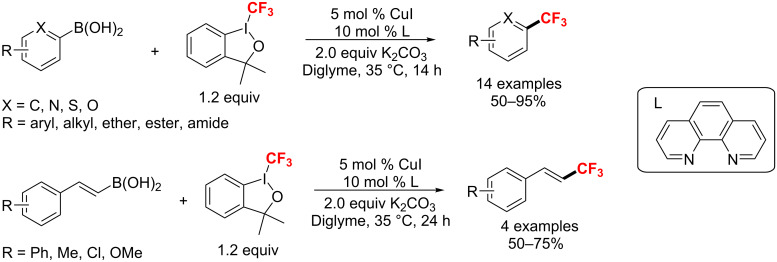
CuI-catalyzed trifluoromethylation of aryl- and alkenylboronic acids.

**Copper-catalyzed trifluoromethylation of aryl- and alkenylboronic acids:** In 2011, Liu and Shen [128] developed a CuI-catalyzed method for the trifluoromethylation of aryl- and alkenylboronic acids with Togni’s reagent (Scheme 68). A range of different substrates gave the corresponding trifluoromethylated (hetero)arenes in good to excellent yields.

**Scheme 69:**
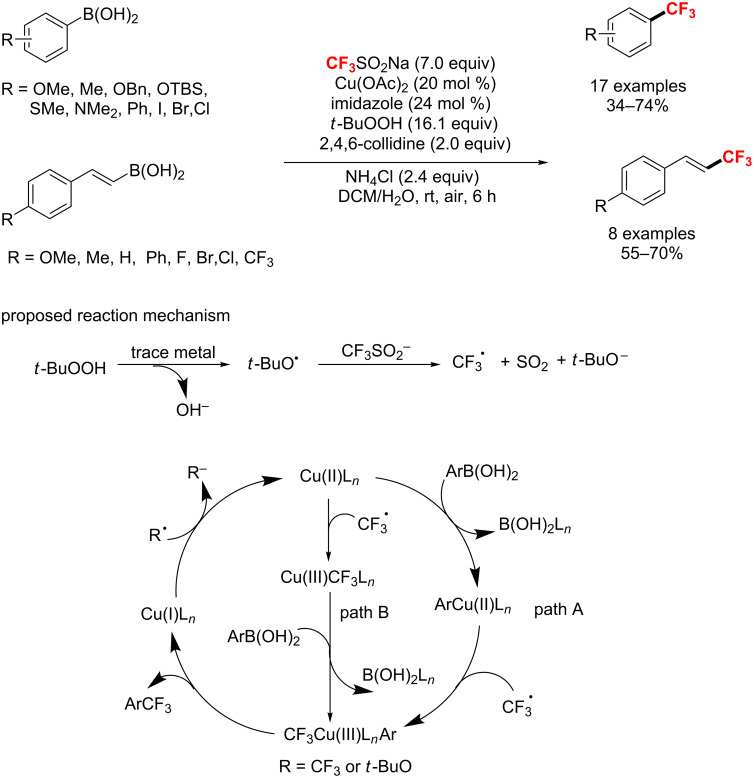
Cu-catalyzed trifluoromethylation of aryl- and vinylboronic acids.

Also, in 2012, Beller and co-workers [129] described a copper-catalyzed trifluoromethylation of aryl- and vinylboronic acids with the generation of CF_3_-radicals at room temperature. The mild reaction conditions allowed a wide variety of functional groups to be tolerated, though a large quantity of TBHP was required (Scheme 69). Notably, the authors proposed two mechanistic pathways for this trifluoromethylation reaction. The difference between path A and path B is that the sequence of CF_3_ radicals and aryl- and vinylboronic acids is reversed. In addition, the CF_3_ radical is generated from the reaction of TBHP with NaSO_2_CF_3_.

**Scheme 70:**
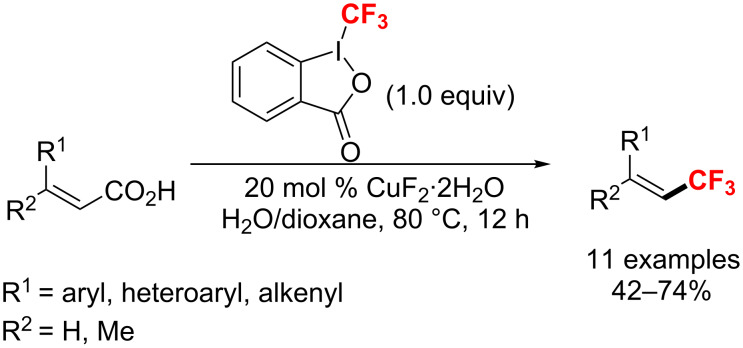
Copper-catalyzed trifluoromethylation of α,β-unsaturated carboxylic acids.

**Copper-catalyzed trifluoromethylation of alkenes:** The method described by Hu [130] was applied to the trifluoromethylation of a wide range of α,β-unsaturated carboxylic acids through CuF_2_-catalyzed decarboxylative fluoroalkylation with high yields and excellent *E*/*Z* ratio (Scheme 70).

**Scheme 71:**
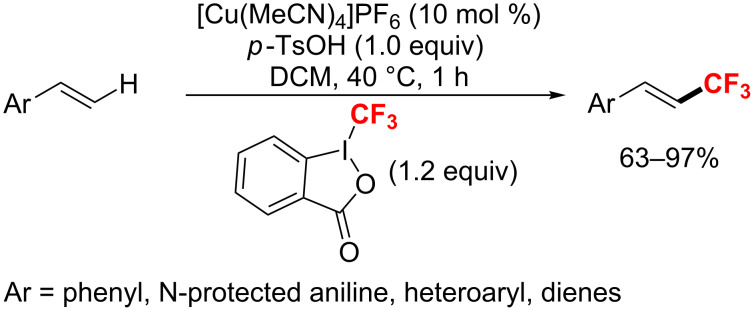
Formation of C(sp^2^)–CF_3_ bond catalyzed by copper(I) complex.

Additionally, a copper(I)-catalyzed trifluoromethylation of alkenes was disclosed by Sodeoka and co-workers in 2012 [131]. The reaction was carried out with Togni’s reagent as the CF_3_ source and TsOH as a Brønsted acid in CH_2_Cl_2_ at 40 °C (Scheme 71). Notably, trifluoromethylstyrenes were formed through further transformations of the oxytrifluoromethylated products with high efficiency.

**Scheme 72:**
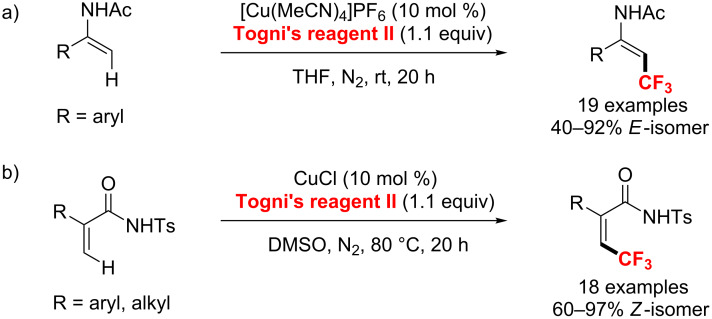
Loh’s Cu(I)-catalyzed trifluoromethylation of enamides and electron-deficient alkenes.

In the same year, Loh’s group [132] used the same copper catalyst and Togni’s reagent to achieve the trifluoromethylation of enamides in good yields at room temperature (Scheme 72a). Meanwhile, this reaction exhibited excellent stereoselectivity towards the *E*-isomer. One year later, the same group [133] extended this approach to the directing-group-assisted copper-catalyzed trifluoromethylation of electron-deficient alkenes (Scheme 72b). Moreover, α-aryl and α-alkyl-substituted acrylate derivatives could be used as substrates to form the C(sp^2^)–CF_3_ bond with a complete *Z-*selectivity. A radical species participated in the reaction’s catalytic cycle.

**Scheme 73:**
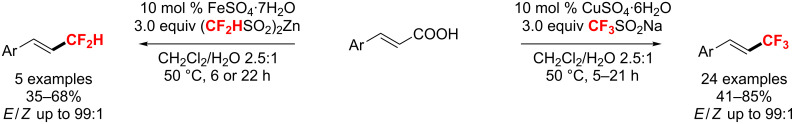
Copper and iron-catalyzed decarboxylative tri- and difluoromethylation.

In 2013, the group of Liu [134] described a copper-catalyzed decarboxylative trifluoromethylation of α,β-unsaturated carboxylic acids with CF_3_SO_2_Na. This method was applied to a wide range of α,β-unsaturated carboxylic acids. Meanwhile, a similar radical process for the difluoromethylation of aryl-substituted acrylic acids was also achieved by Liu and co-workers. The HCF_2_-substituted *E*-alkenes were finally obtained with iron catalysis and zinc difluoromethanesulfinate ((CF_2_HSO_2_)_2_Zn, Baran reagent). Also, the authors proved that the formation of the C_vinyl_–CF_3_/C_vinyl_–CF_2_H bonds followed a radical addition–elimination process (Scheme 73).

**Scheme 74:**
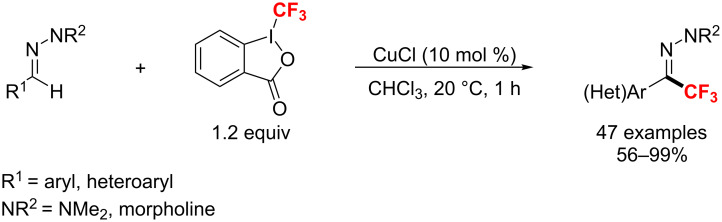
Cu-catalyzed trifluoromethylation of hydrazones developed by Bouyssi.

Subsequently, Bouyssi and co-workers [135–137] used Togni’s reagent to conduct the trifluoromethylation of (hetero)aromatic aldehydes or corresponding *N*,*N*-dialkylhydrazones with CuCl as the catalyst at room temperature (Scheme 74). These reactions showed a broad substrate scope and good functional group compatibility with up to 99% yield.

**Scheme 75:**
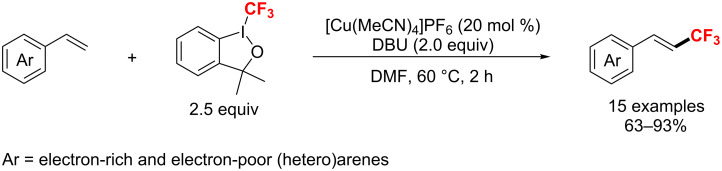
Cu(I)-catalyzed trifluoromethylation of terminal alkenes.

In 2013, a simple and effective copper-catalyzed approach for the construction of C_vinyl_–CF_3_ bonds without using pre-functionalized substrates was reported by Xiao et al. (Scheme 75) [138]. The process proceeded smoothly to give the trifluoromethylated alkenes in good to excellent yields via a radical mechanism.

**Scheme 76:**
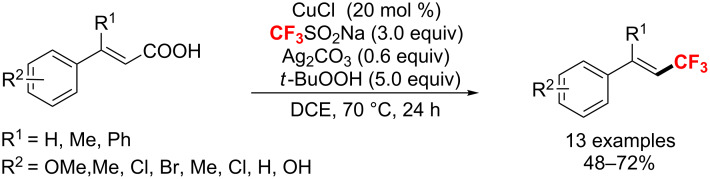
Cu/Ag-catalyzed decarboxylative trifluoromethylation of cinnamic acids.

Additionally, Duan and co-workers [139] discovered a copper/silver-catalyzed decarboxylative trifluoromethylation of α,β-unsaturated carboxylic acids with CF_3_SO_2_Na. This reaction proceeded efficiently for a wide range of alkyl and aryl-substituted α,β-unsaturated carboxylic acids derivatives with excellent *E*/*Z* selectivity (Scheme 76). It's worth mentioning that the addition of Ag_2_CO_3_ additives was crucial for promoting the decarboxylation of α,β-unsaturated carboxylic acids.

**Scheme 77:**
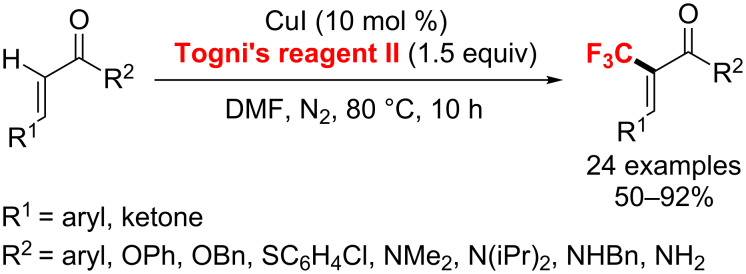
Copper-catalyzed direct alkenyl C–H trifluoromethylation.

In 2014, a Cu(I/II)-catalyzed α-trifluoromethylation of α,β-unsaturated carbonyl compounds were unfolded by the Bi group (Scheme 77) [140]. The reaction was applied to a broad range of carbonyl compounds, including enones, α,β-unsaturated esters, thioesters, and amides. Notably, the authors obtained products with stable *E*-configuration through a SET process.

**Scheme 78:**
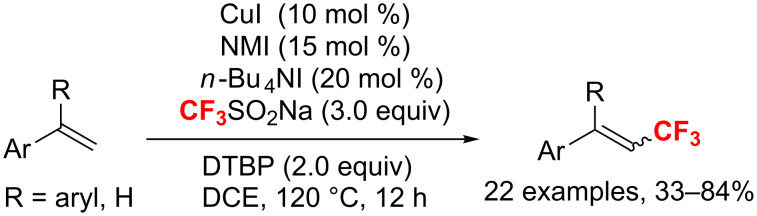
Copper(I/II)-catalyzed direct trifluoromethylation of styrene derivatives.

In 2017, Loh and co-workers [141] introduced a Cu(I/II)-catalyzed C_vinyl_–H trifluoromethylation of a variety of styrene derivatives. This process was achieved by using 1-methylimidazole (NMI) as ligand and tetrabutylammonium iodide (TBAI) as an additive (Scheme 78). Mechanistic studies revealed that this reaction probably proceeds through a radical pathway.

**Scheme 79:**
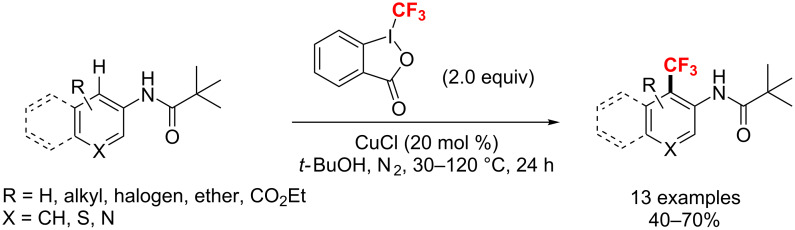
Regioselective trifluoromethylation of pivalamido arenes and heteroarenes.

**Copper-catalyzed trifluoromethylation of arenes and heteroarenes:** In 2013, Xi et al. [142] reported a CuCl-catalyzed direct trifluoromethylation of sp^2^ C–H bonds with Togni reagent (Scheme 79). Also, phenyl, thiophene, and pyridine derivatives achieved regioselectively trifluoromethylation with *N*-pivalamide as a directing group. The authors also proposed a possible radical pathway for this reaction. The final trifluoromethylated compounds were generated from pivalamido arenes and heteroarenes with the CF_3_ radical through a Cu(I/II) catalytic cycle.

**Scheme 80:**
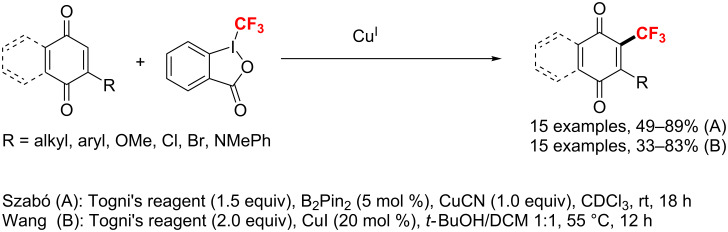
Synthesis of trifluoromethylquinones in the presence of copper(I).

In 2013, the Szabó [143] and Wang group [144] described the copper-mediated C–H trifluoromethylation of quinones with Togni’s reagent. Szabó utilized a stoichiometric amount of CuCN combined with catalytic bis(pinacolato)diboron, whereas Wang applied a stoichiometric amount of CuI. Notably, both groups proved a mechanism involving the formation of a CF_3_ radical with copper(I) acting as a one-electron reducing agent (Scheme 80).

**Scheme 81:**
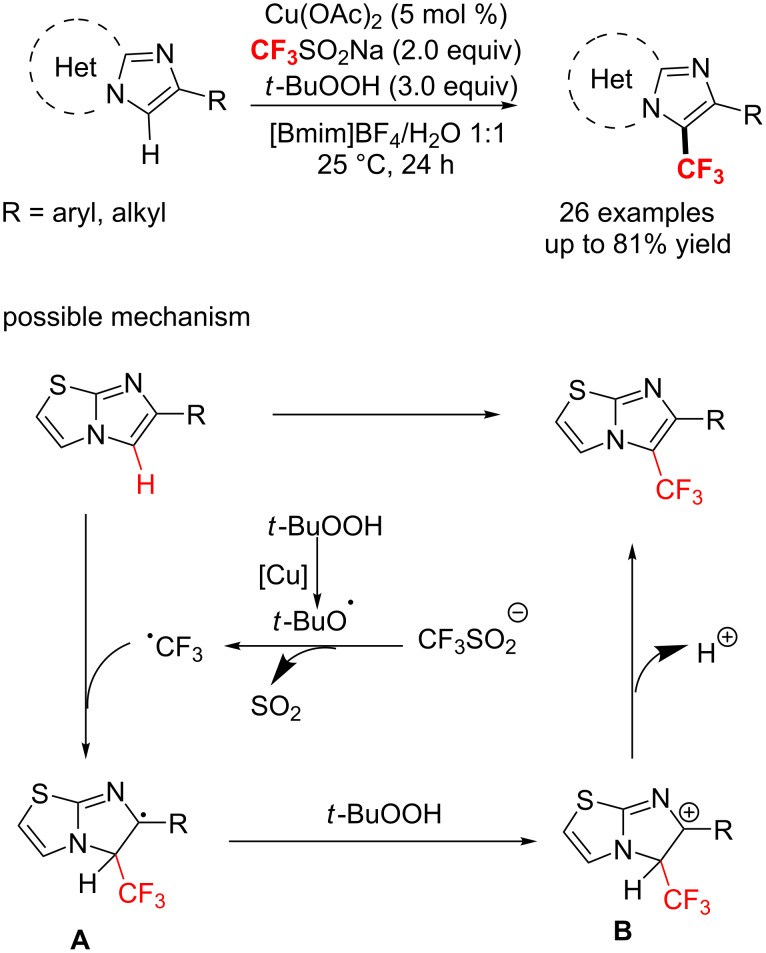
Oxidative trifluoromethylation of imidazoheterocycles in ionic liquid/water.

With catalytic cupric acetate and TBHP, the group of Tang [145] developed a green strategy for the trifluoromethylation of imidazoheterocycles with a recyclable mixed medium of 1-butyl-3-methylimidazolium tetrafluoroborate ([Bmim]BF_4_) and water (Scheme 81). By following this method, diverse trifluoromethylated imidazoheterocycles were obtained in up to 80% yield. The method features a green and recyclable solvent, mild reaction conditions (room temperature) and excellent functional group tolerance. In this instance, the copper catalyst may only promote the generation of the *tert*-butoxyl radical from TBHP. The oxidation of the intermediate A with *t*-BuOOH produces a carbocation B, followed by an oxidative dehydrogenation process to afford the target product.

**Scheme 82:**
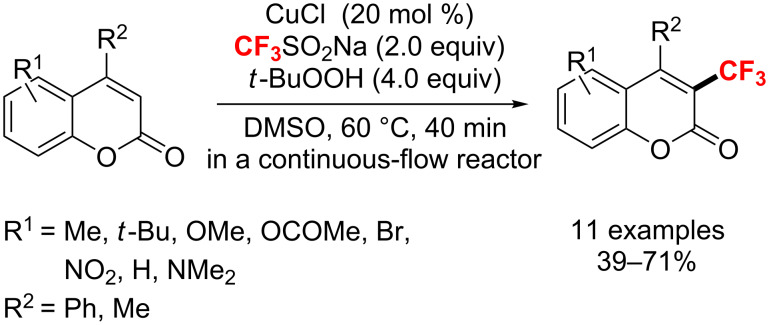
A mild and fast continuous-flow trifluoromethylation of coumarins using a CuI/CF_3_SO_2_Na/TBHP system.

Also in 2015, Li and co-workers [146] developed a mild and fast Cu(I/II)-catalyzed trifluoromethylation procedure to obtain 3-trifluoromethylcoumarins. The reaction was carried out with a CuCl/CF_3_SO_2_Na/TBHP system under continuous-flow conditions, affording the corresponding products with wide substrate tolerance in moderate to good yields (Scheme 82).

**Scheme 83:**
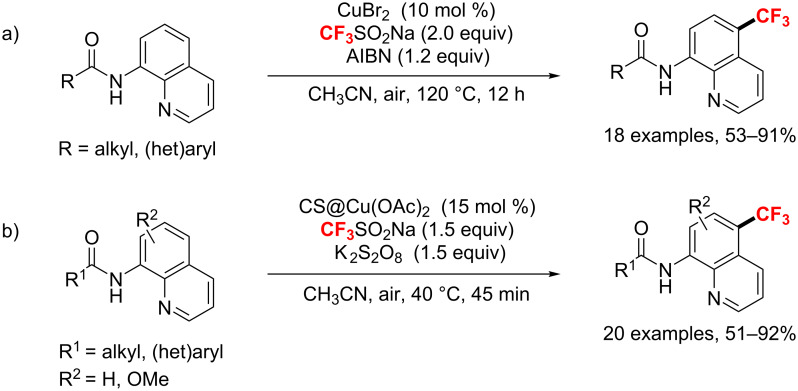
Copper-catalyzed oxidative trifluoromethylation of various 8-aminoquinolines.

After one year, the group of Cai [147] presented a Cu(II)-catalyzed 8-amido chelation-induced regioselective C5-trifluoromethylation of quinolines (Scheme 83a). With CuBr_2_ as a catalyst and azobisisobutyronitrile (AIBN) as an oxidant, a wide range of functional groups were well tolerated to provide the products in moderate to excellent yields. Simultaneously, Zhang and co-workers [148] described a similar, milder regioselective C–H trifluoromethylation of 8-aminoquinolines by using a chitosan-based heterogeneous copper catalyst (CS@Cu(OAc)_2_, CS = chitosan) (Scheme 83b).

**Scheme 84:**
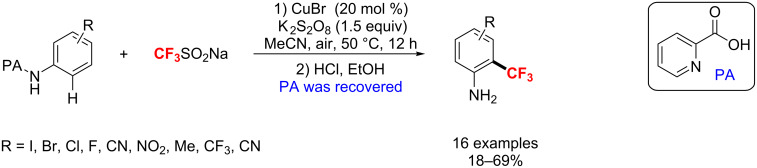
PA-directed copper-catalyzed trifluoromethylation of anilines.

Recently, a picolinamide (PA)-directed method for the Cu-catalyzed trifluoromethylation of anilines was described by the group of Zhang [149]. The trifluoromethyl group was installed at the *ortho* position of the substrate, yielding 2-(trifluoromethyl)aniline derivatives in moderate to good yields (Scheme 84). Notably, the directing group could be recovered in excellent yields and this approach provided a new way for the efficient synthesis of floctafenine via a single-electron-transfer mechanism.

**Scheme 85:**
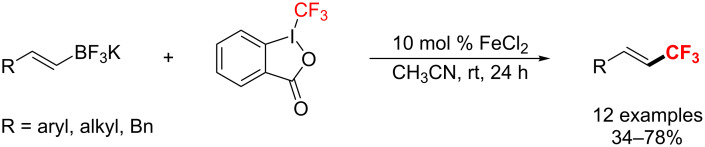
Trifluoromethylation of potassium vinyltrifluoroborates catalyzed by Fe(II).

**Vinyl C–CF_3_ bond formation using Fe, Ir, Ru, and Ag catalysts:** In 2012, Buchwald and co-workers [150] unfolded an iron(II)-catalyzed trifluoromethylation of potassium vinyltrifluoroborates at room temperature (Scheme 85). With this approach, 2-arylvinyl substrates, in particular, furnished the products in good yields and excellent *E*/*Z* ratios (*E*/*Z* > 95.5%).

**Scheme 86:**
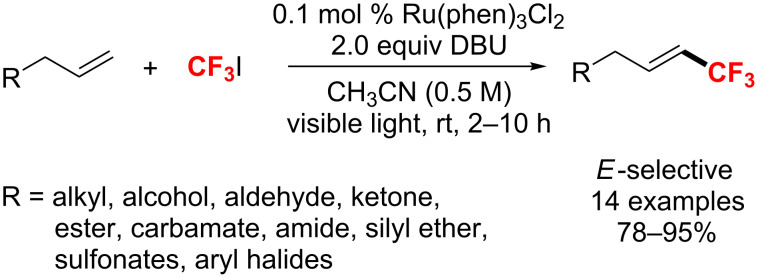
Alkenyl trifluoromethylation catalyzed by Ru(phen)_3_Cl_2_ as photocatalyst.

Also, Cho and co-workers [151] reported a direct method for an alkenyl trifluoromethylation employing a Ru photocatalyst. The method used CF_3_I as a CF_3_ radical source and 1,8-diazabicyclo[5.4.0]undec-7-ene (DBU) as the base (Scheme 86). Under these mild reaction conditions, the trifluoromethylation of a wide range of alkenes shows high functional-group tolerance with a low catalyst loading. Moreover, compared with other alkenes, this process works especially well for terminal alkenes.

**Scheme 87:**
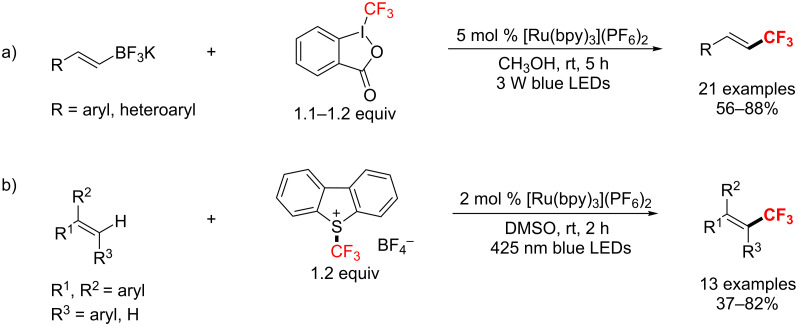
Ru-catalyzed trifluoromethylation of alkenes by Akita’s group.

In 2013, Akita’s group [152] developed a radical-mediated trifluoromethylation of vinyltrifluoroborates promoted by the photoredox catalyst [Ru(bpy)_3_](PF_6_)_2_ under visible light irradiation (Scheme 87a). The trifluoromethylated alkenes were obtained in up to 95% yield. One year later, the same group [153] presented a procedure for trifluoromethylation of multisubstituted alkenes with a different CF_3_ source, Umemoto’s reagent (Scheme 87b). Additionally, this reaction could be extended to double trifluoromethylation.

**Scheme 88:**
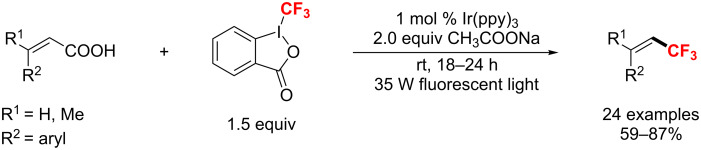
Ir-catalyzed C_vinyl_–CF_3_ bond formation of α,β-unsaturated carboxylic acids.

In 2014, a visible-light-induced decarboxylative trifluoromethylation of α,β-unsaturated carboxylic acids by using [Ir(ppy)_3_] as a photoredox catalyst was explored by Zhu and co-workers (Scheme 88) [154]. Notably, this procedure employed only 1 mol % catalyst loading to achieve an excellent reactivity and *E*/*Z* stereoselectivity at room temperature.

**Scheme 89:**
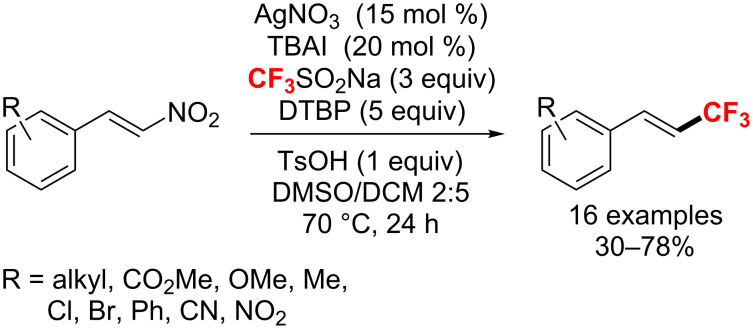
Ag(I)-catalyzed denitrative trifluoromethylation of β-nitrostyrenes.

In 2016, Duan and co-workers [155] disclosed a Ag(I)-catalyzed denitration/trifluoromethylation of β-nitrostyrenes with CF_3_SO_2_Na, which employed a large excess of di-*tert*-butyl peroxide (DTBP) as the oxidant and tetrabutylammonium iodide (TBAI) as phase-transfer catalyst (Scheme 89). Notably, only (*E*)-isomers of the products were obtained in moderate to high yields.

**Scheme 90:**
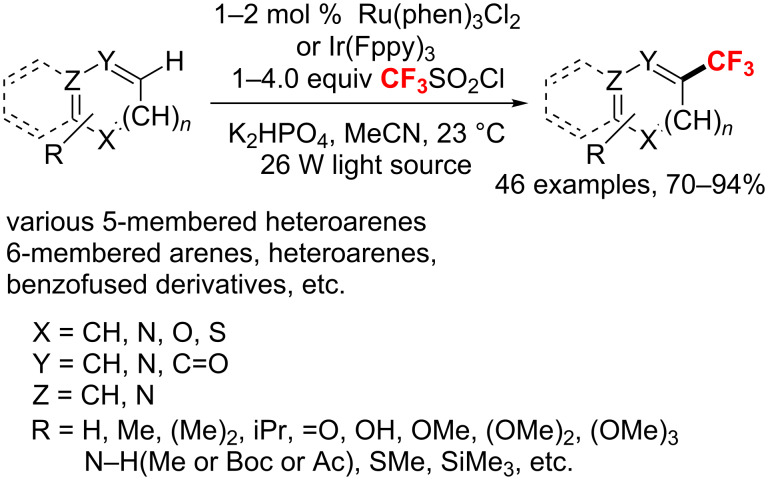
Photocatalyzed direct trifluoromethylation of aryl and heteroaryl C–H bonds.

**Various transition-metal-catalyzed direct C–H bond trifluoromethylation of arenes and heteroarenes:** In 2011, the group of MacMillan [156] reported a simple approach for the direct trifluoromethylation of unactivated arenes and heteroarenes through a radical-mediated mechanism (Scheme 90). Under exposure to 26 W fluorescent light, this process proceeded well in the presence of triflyl chloride and different photocatalysts depending on the substrate’s nature, i.e., Ru(phen)_3_Cl_2_ for 5-membered heterocycles, Ir(Fppy)_3_ for 6-membered arenes and heterocycles. It is worth mentioning, that triflyl chloride provides a cheap and easy to handle CF_3_ source.

**Scheme 91:**
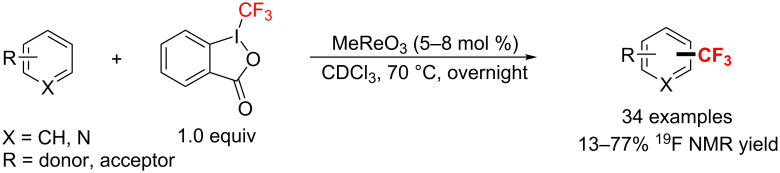
Rhenium (MTO)-catalyzed direct trifluoromethylation of aromatic substrates.

A mild and simple electrophilic trifluoromethylation of various aromatic and heteroaromatic compounds was disclosed by the Togni group [157] in 2012. The authors used methyltrioxorhenium (MTO) as the catalyst (Scheme 91). Notably, the direct aromatic trifluoromethylation tolerates a broad substrate scope, however, is limited to electron-rich substrates.

**Scheme 92:**
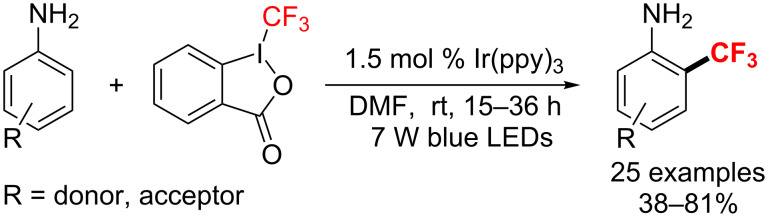
Trifluoromethylation of unprotected anilines under [Ir(ppy)_3_] catalyst.

In 2014, Ma et al. [158] developed the first visible-light-promoted radical trifluoromethylation of unprotected anilines. With [Ir(ppy)_3_] and Togni’s reagent, the method afforded various fluorine-containing molecules and heterocyclic compounds at room temperature (Scheme 92).

**Scheme 93:**
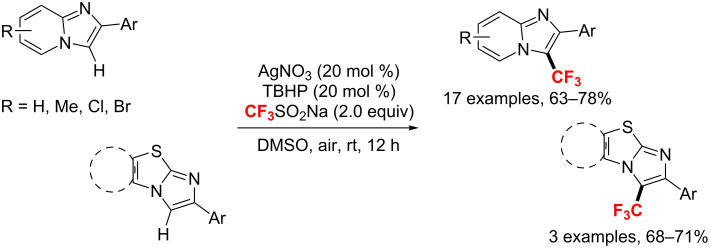
Oxidative trifluoromethylation of imidazopyridines and imidazoheterocycles.

In 2015, the group of Hajra [159] described a method for the direct trifluoromethylation of imidazopyridines and other imidazoheterocycles. The CF_3_SO_2_Na/*t*-BuOOH/Ag system enables accomplishing the reaction at room temperature under ambient air (Scheme 93).

**Scheme 94:**
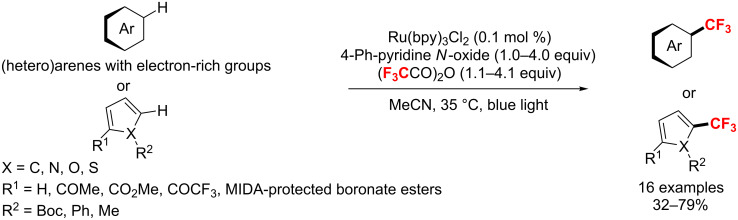
Ruthenium-catalyzed trifluoromethylation of (hetero)arenes with trifluoroacetic anhydride.

A direct trifluoromethylation of (hetero)arenes in the presence of only 0.1 mol % [Ru(bpy)_3_]Cl_2_ as catalyst was reported by the Stephenson group in 2016 [160]. Notably, the authors utilized pyridine *N*-oxide derivatives in concert with trifluoroacetic anhydride to facilitate this process (Scheme 94). Moreover, the method has been successfully extended on a kilogram scale.

**Scheme 95:**
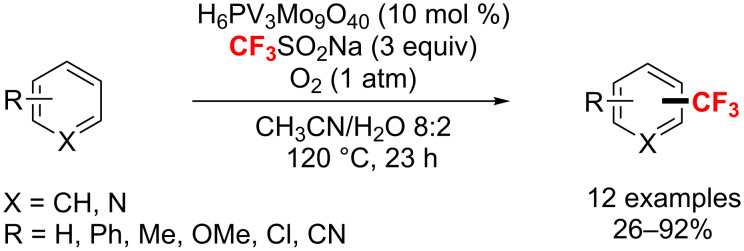
Phosphovanadomolybdic acid-catalyzed direct C–H trifluoromethylation.

One year later Mizuno’s group [161] introduced a direct C–H trifluoromethylation of (hetero)arenes with O_2_ as the terminal oxidant in the presence of catalytic amounts of phosphovanadomolybdic acids (Scheme 95). The reaction tolerated diverse (hetero)arenes to afford the corresponding trifluoromethylated products via a radical pathway in 26–92% yields.

**Scheme 96:**
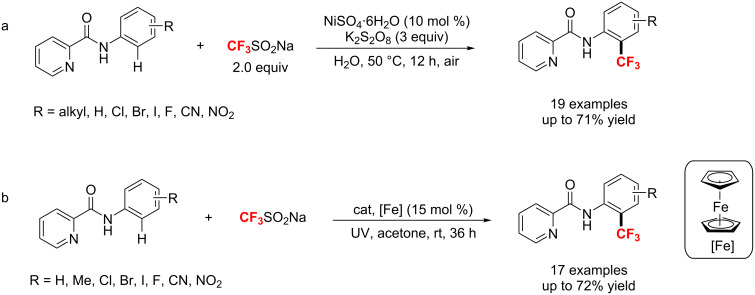
Picolinamide-assisted *ortho*-trifluoromethylation of arylamines.

In 2017, Zhang and co-workers [162] were the first who reported a nickel(II)-catalyzed and picolinamide-assisted site-selective C–H bond trifluoromethylation of arylamines in water (Scheme 96a). This strategy displays several advantages: 1) inexpensive nickel catalyst, 2) recyclable catalyst, 3) aqueous phase reaction, and 4) high site selectivity. Only one year later, the group of Xia optimized this approach and established a convenient, oxidant-free protocol for the *ortho-*trifluoromethylation of arylamine under ultraviolet irradiation (Scheme 96b) [163].

**Scheme 97:**
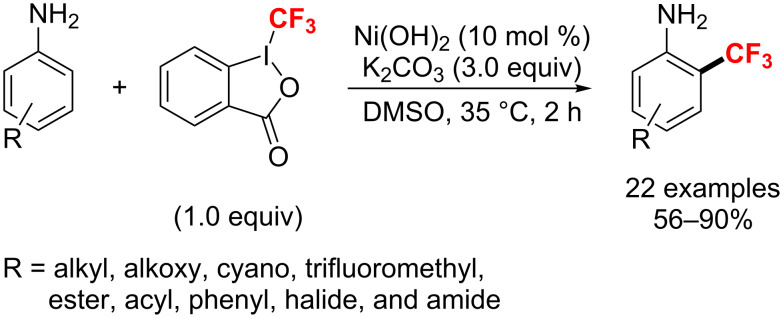
A nickel-catalyzed C–H trifluoromethylation of free anilines.

In 2018, Wu and co-workers [164] introduced a one-step strategy for the synthesis of trifluoromethylated free anilines using Togni’s reagent using a nickel-catalyzed C–H trifluoromethylation. Moreover, free anilines with a variety of functional groups were trifluoromethylated under the mild reaction conditions in up to 90% yield (Scheme 97).

#### C(sp)–CF_3_ bond formation

**Scheme 98:**
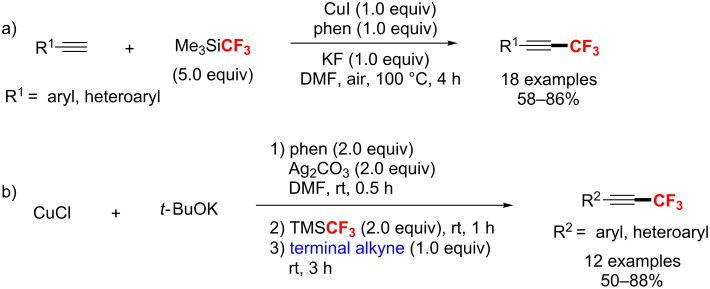
Cu-mediated trifluoromethylation of terminal alkynes reported by Qing.

In 2010, Qing’s group [165] reported the first example of a copper-mediated trifluoromethylation of terminal alkynes. Notably, the reaction was carried out with nucleophilic (trifluoromethyl)trimethylsilane (Me_3_SiCF_3_) as a CF_3_ source under air atmosphere (Scheme 98a). Moreover, this protocol was compatible with various terminal alkynes, such as aromatic and aliphatic alkynes, affording the trifluoromethylated alkynes in 47–91% yields. Subsequently, Qing [166] developed an efficient catalytic trifluoromethylation by adding terminal alkynes and Me_3_SiCF_3_ slowly with a syringe pump. Two years later, the same group [167] presented an improved Cu-mediated oxidative trifluoromethylation of aryl and heteroaryl terminal alkynes. In the latter case, the trifluoromethylation proceeded at room temperature by using Ag_2_CO_3_ as an oxidant with a significantly lower amount of TMSCF_3_ (Scheme 98b).

**Scheme 99:**
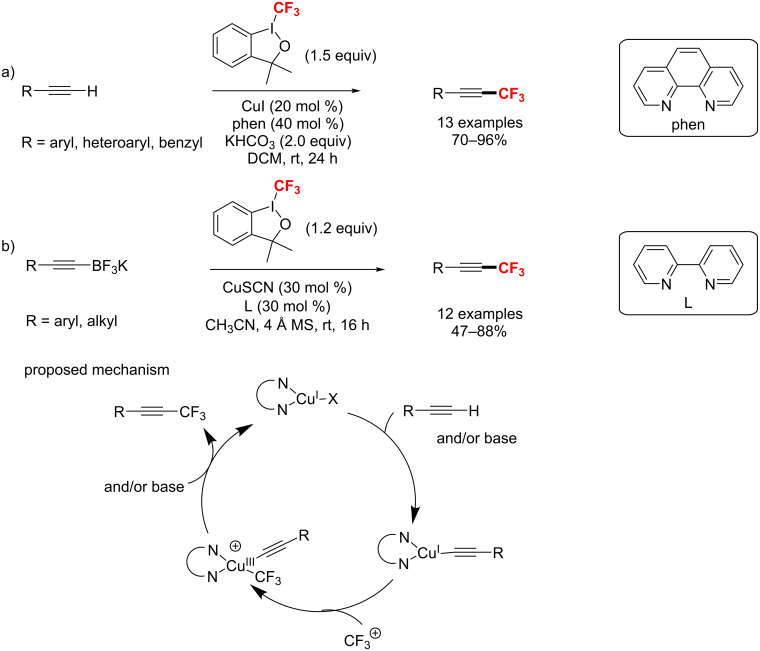
Huang’s C(sp)–H trifluoromethylation using Togni’s reagent.

In 2012, Huang et al. [168] reported a process for trifluoromethylation of terminal alkynes with Togni’s reagent in DCM at room temperature (Scheme 99a). The trifluoromethylated acetylenes were obtained with up to 98% yield via a Cu(I/III) catalytic cycle with CF_3_^+^. As an extension of their work, this group [169] developed the trifluoromethylation of alkynyltrifluoroborates to form trifluoromethylated acetylenes under similar conditions without the addition of bases (Scheme 99b).

**Scheme 100:**
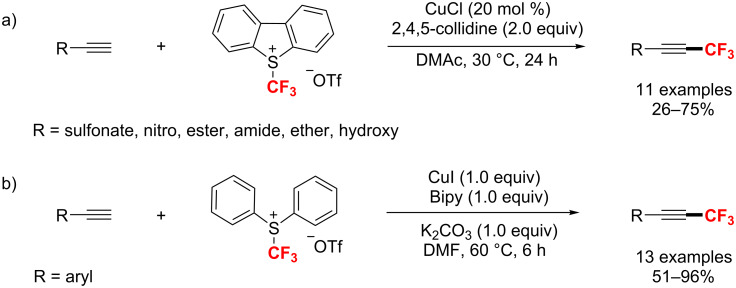
Cu-catalyzed methods for trifluoromethylation with Umemoto’s reagent.

In the same year, the groups of Guo [170] and Xiao [171] also developed a copper(I)-catalyzed trifluoromethylation of terminal alkynes with Umemoto’s reagent as an electrophilic CF_3_ source (Scheme 100a). Compared with the reaction conditions reported by Guo, Xiao’s method was carried out at higher temperature, using similar copper(I) catalysts, but with different ligands (Scheme 100b).

**Scheme 101:**
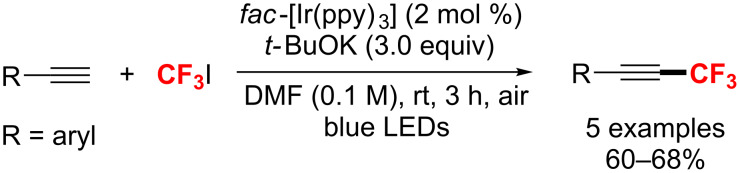
The synthesis of alkynyl-CF_3_ compounds in the presence of *fac*-[Ir(ppy)_3_] under visible-light irradiation.

In 2014, the trifluoromethylation of aromatic alkynes through visible-light photoredox catalysis was described by Cho and co-workers [172]. With *fac*-[Ir(ppy)_3_] as photocatalyst and KO*t*-Bu as a base, the reaction was achieved under blue LED irradiation in moderate yields (Scheme 101). However, this approach was not suitable for aliphatic alkynes.

Compared with the trifluoromethylation and fluorination mentioned above, the methodological research on difluoromethylation, trifluoromethylthiolation and trifluoromethoxylation of organic molecules are quite rare and scattered. Here we summarize the new developments within recent years.

### Difluoromethylation

The introduction of a difluoromethylene (CF_2_) group into organic molecules can significantly improve their metabolic stability and oral bioavailability [3]. Therefore, the difluoroalkylation has become a powerful strategy for regulating the biological activity of organic molecules. It is noteworthy that transition-metal-catalyzed difluoroalkylation is an effective route to obtain these valuable difluoroalkylated backbones. There are four modes of catalytic difluoroalkylation, including nucleophilic difluoroalkylation, electrophilic difluoroalkylation, radical difluoroalkylation, and metal-difluorocarbene coupling (MeDiC) [173]. Finally, a wide range of difluoroalkylated (hetero)arenes [(Het)Ar-CF_2_R, R = PO(OEt)_2_, CO_2_Et, CONR^1^R^2^, COR^1^, (Het)Ar, alkenyl, alkynyl, alkyl, H] and alkenes were obtained with excellent functional group tolerance.

**Scheme 102:**
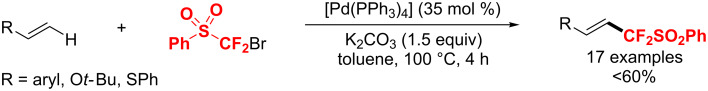
Pd-catalyzed Heck reaction reported by Reutrakul.

In 2012, the Reutrakul group [174] firstly reported a Pd-catalyzed Heck-type reaction of [(bromodifluoromethyl)sulfonyl]benzene with styrene derivatives (Scheme 102). Notably, the reaction shows a broad substrate scope, including a variety of styrene derivatives, vinyl ethers, vinyl sulfides, and a few heteroaromatic substrates.

**Scheme 103:**
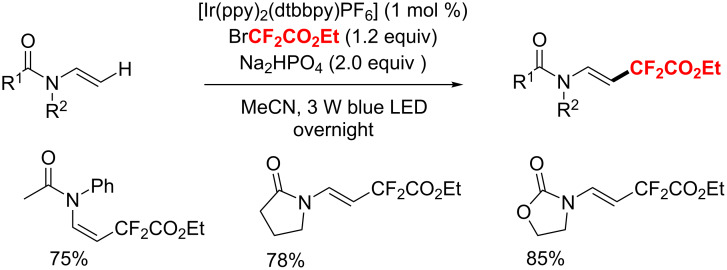
Difluoromethylation of enamides and ene-carbamates.

In the same year, Yu and co-workers [175] developed an iridium-catalyzed direct C–H functionalization of enamides and ene-carbamates with BrCF_2_CO_2_Et under visible-light photoredox conditions (Scheme 103). This method shows excellent yields and a wide substrate scope.

**Scheme 104:**
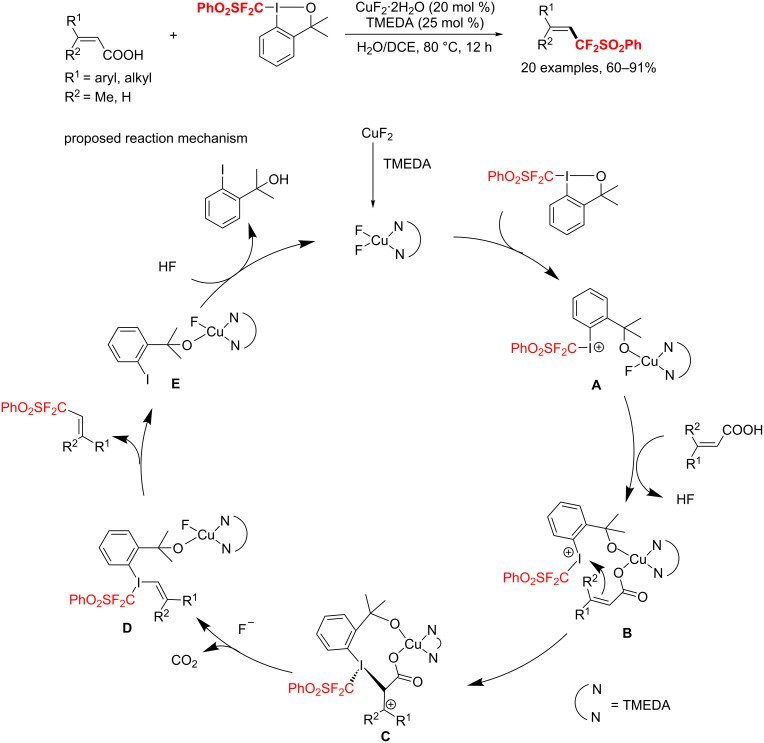
Difluoromethylation of α,β-unsaturated carboxylic acids.

Moreover, Hu et al. [130] established a copper-catalyzed (phenylsulfonyl)-difluoromethylation of α,β-unsaturated carboxylic acids with excellent *E*/*Z* selectivity (Scheme 104). Notably, the Lewis acid (CuF_2_·2H_2_O) was used to enhance the electrophilicity of the Togni’s reagent and to promote the decarboxylation of the carboxylic acids. The authors proposed that, under these conditions, the Togni’s reagent may undergo a Cu-catalyzed bond cleavage to produce the highly electrophilic iodonium salt A, which then coordinates to the carboxylic acid functionality to generate the intermediate **B**. The latter then undergoes – through an intramolecular reaction – decarboxylation and reductive elimination to afford the species **E** and the desired product. Finally, species **E** reacts with HF regenerating the catalyst.

**Scheme 105:**
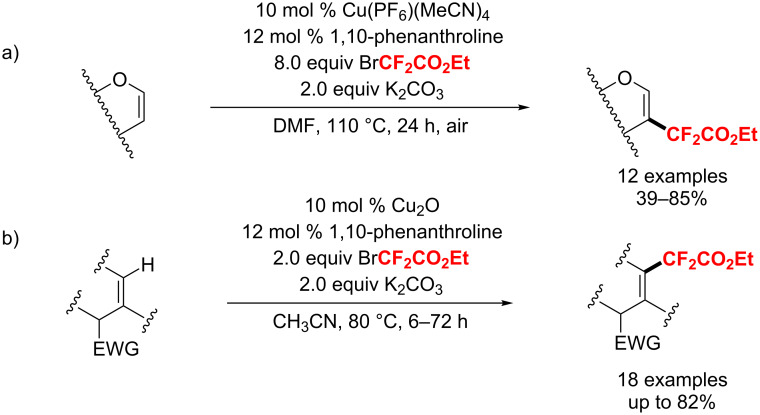
Copper-catalyzed direct C(sp^2^)–H difluoroacetylation reported by Pannecoucke and co-workers.

In 2013, Pannecoucke and co-workers [176] developed a copper-catalyzed regioselective difluoroacetylation of dihydropyrans and glycals on the C-2 position. Notably, the corresponding products were obtained through a Cu(I/III) catalytic cycle without the involvement of radicals (Scheme 105a). Hence, in 2014, the same group extended this method to the olefinic difluoroacetylation of enamides [177]. In this reaction, they obtained the β-difluoroester-substituted enamides under operationally simple and mild conditions. Also, the method has a broad substrate scope, including cyclic and acyclic enamides (Scheme 105b).

**Scheme 106:**
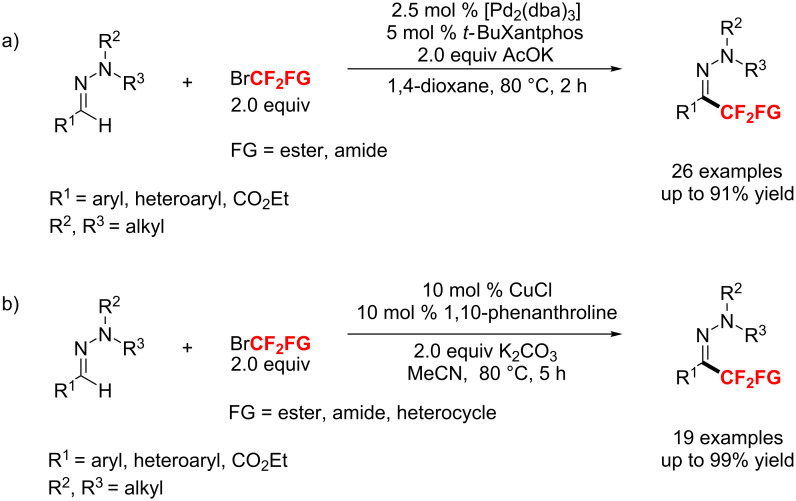
Difluoroalkylation of aldehyde-derived hydrazones with functionalized difluoromethyl bromides.

In 2016, a Pd-catalyzed direct difluoroalkylation of aldehyde hydrazones with functionalized difluoromethyl bromides was described by Monteiro’s group (Scheme 106a) [178]. The bromodifluoromethylated compounds are effective reagents for the difluoromethylation of aldehyde-derived hydrazones to the corresponding difluoromethyl ketone hydrazones. However, this strategy relies on the use of an expensive palladium/ligand catalyst system that makes it less attractive and practical. Subsequently, the same group [179] found that CuCl could also effectively catalyze the difluoromethylation of hydrazones. This method provided an efficient, cost-effective protocol for the multigram-scale preparation of functionalized difluoromethylketone hydrazines (Scheme 106b).

**Scheme 107:**
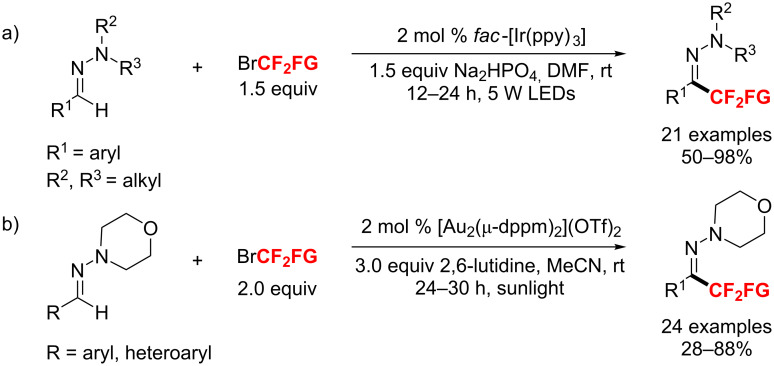
Photoredox-catalyzed C–H difluoroalkylation of aldehyde-derived hydrazones.

Compared with Monteiro’s approaches, Zhu and co-workers [180] were the first who developed a visible-light-induced direct C–H-bond difluoroalkylation of aldehyde-derived hydrazones (Scheme 107a). Importantly, this unprecedented photoredox protocol is enabled by a novel aminyl radical/polar crossover mechanism. Meanwhile, a first gold-catalyzed photoredox difluoroalkylation of aromatic aldehyde hydrazones under sunlight was reported by Hashmi’s group (Scheme 107b) [181]. Both methods smoothly work at room temperature affording the products with modest to excellent yields.

**Scheme 108:**
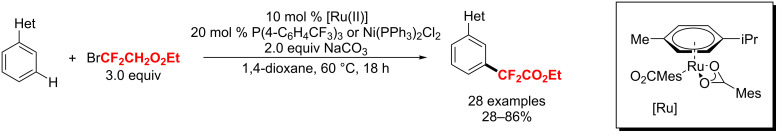
Synergistic ruthenium(II)-catalyzed C–H difluoromethylation reported by Ackermann.

One year later, Ackermann and co-workers [182] presented a ruthenium(II)-catalyzed *meta*-selective C–H difluoromethylation with the cooperation of phosphine and carboxylate. This protocol is compatible with a variety of functional groups, such as pyridyl, pyrimidyl, pyrazolyl, and even purinyl assistance (Scheme 108).

**Scheme 109:**
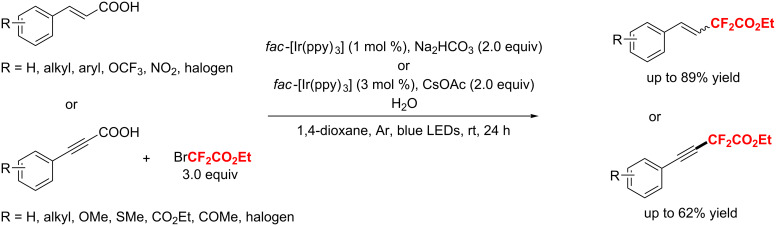
Visible-light photocatalytic decarboxylation of α,β-unsaturated carboxylic acids.

A visible-light photocatalytic decarboxylation strategy for the synthesis of difluoromethylated styrenes with *fac*-Ir(ppy)_3_ and BrCF_2_CO_2_Et was developed by Noël et al. in 2017 [183]. Herein, *meta* and *para*-substituted cinnamic acids afforded the expected *E*-isomers, while *ortho*-substituted cinnamic acids selectively provided the less stable *Z*-product. Notably, the conversion of the *Z*-isomer into the *E*-isomer was achieved by controlling the reaction time accurately. Furthermore, arylpropiolic acids could also be decarboxylative difluoromethylated by this method (Scheme 109).

**Scheme 110:**
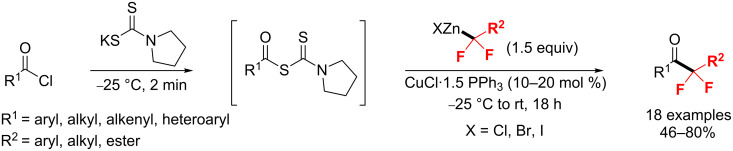
Synthesis of difluorinated ketones via *S*-alkyl dithiocarbamates obtained from acyl chlorides and potassium dithiocarbamate.

Meanwhile, the group of Dilman [184] developed a method for the synthesis of difluorinated ketones with *gem*-difluorinated organozinc reagents (Scheme 110). Firstly, acyl chlorides reacted with potassium dithiocarbamate to generate *S*-acyl dithiocarbamates. Subsequently, the so-obtained dithiocarbamates were coupled with organozinc to produce the desired difluorinated ketones.

**Scheme 111:**
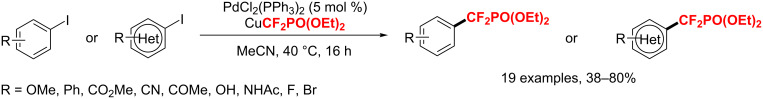
Synthesis of aryl and heteroaryl difluoromethylated phosphonates.

Additionally, Poisson and co-workers [185] developed a simple and efficient way to access various aryl and heteroaryl difluoromethylated phosphonates under mild conditions. The reaction proceeds smoothly with CuCF_2_PO(OEt)_2_ and a palladium catalyst in MeCN (Scheme 111). This transformation enabled the functionalization of various less reactive substrates, such as phenol, boronate, ketones, nitriles, esters, etc.

**Scheme 112:**
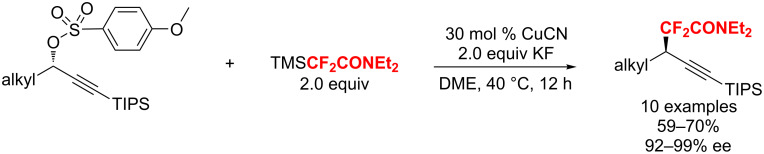
Difluoroalkylation of secondary propargyl sulfonates using Cu as the catalyst.

Notably, the above-mentioned copper-catalyzed highly stereoselective trifluoromethylation reaction of secondary propargyl sulfonates developed by Zhang [116] could also be extended to stereospecific propargylic difluoroalkylation (Scheme 112). In this reaction trimethylsilyldifluoroamide (TMSCF_2_CONEt_2_) is chosen as the difluoroalkylating reagent and proceeds under mild reaction conditions with high regioselectivity and stereospecificity (ee up to 99%).

**Scheme 113:**
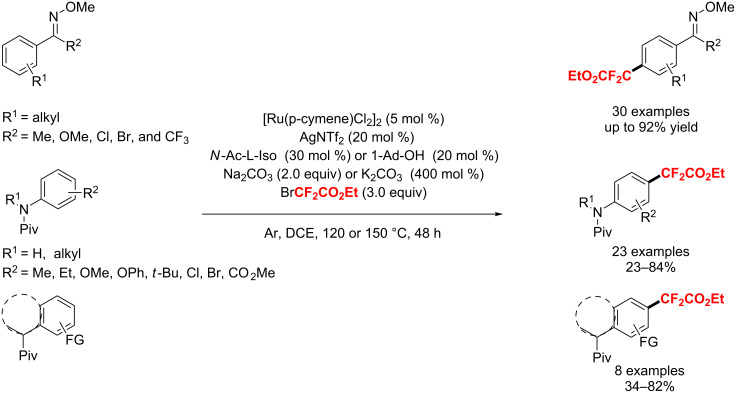
Ru(II)-mediated *para*-selective difluoromethylation of anilides and their derivatives.

In 2018, Zhao et al. [186–187] disclosed a ruthenium(II)-enabled *para*-selective C–H difluoromethylation of ketoxime ethers, anilides, indolines and tetrahydroquinolines (Scheme 113). The protocol is compatible with various functional groups, furnishing the *para*-difluoromethylated products in moderate to good yields. Moreover, chelation-assisted cycloruthenation plays a key role in the selective activation of *para*-C_Ar_–H bonds.

**Scheme 114:**
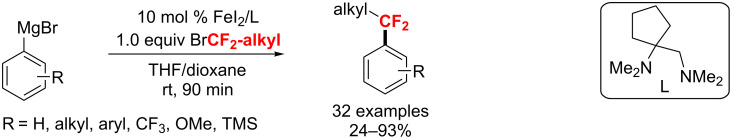
Bulky diamine ligand promoted cross-coupling of difluoroalkyl bromides.

Subsequently, the Zhang group [188] disclosed an iron-catalyzed cross-coupling of a wide range of arylmagnesium and difluoroalkyl bromides with modified *N*,*N*,*N’*,*N’*-tetramethyl-ethane-1,2-diamine (TMEDA) as a ligand. Notably, this bulky diamine is critical to improve the catalytic efficiency and suppress the side reaction of defluorination (Scheme 114).

**Scheme 115:**
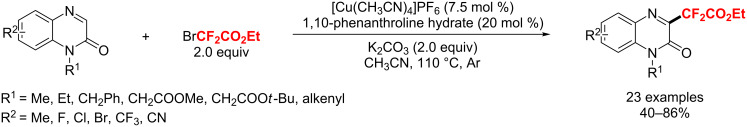
Copper-catalyzed C3–H difluoroacetylation of quinoxalinones.

Recently, a synthetic method for difluoroacetylated quinoxalin-2(1*H*)-one derivatives was presented by the same group [189]. The direct difluoroacetylation of diverse quinoxalinones with a wide range of functional groups proceeded regioselectively at the C-3 position with ethyl bromodifluoroacetate under copper catalysis (Scheme 115).

### Trifluoromethylthiolation

In the past few years, many methods for the direct introduction of trifluoromethylthio groups into organic compounds have been reported. Depending on the type of the trifluoromethylthiolating reagent used in the chemical conversion, the methods can also be classified into three classes: radical, nucleophilic and electrophilic trifluoromethylthiolation. Some reviews in this area focused on several aspects such as the syntheses of aromatic and heterocyclic perfluoroalkyl sulfides [190], direct trifluoromethylthiolation reactions [191–194], sulfur-based fluorination and fluoroalkylation reagents [195], trifluoromethylthio cation-donating ability (Tt^+^DA) [196], or synthetic methods leading to compounds containing CF_3_–S units [197]. Herein, based on the transition-metal catalysis, recent research advances in these types of synthetic methods are described in this section of this review.

**Scheme 116:**
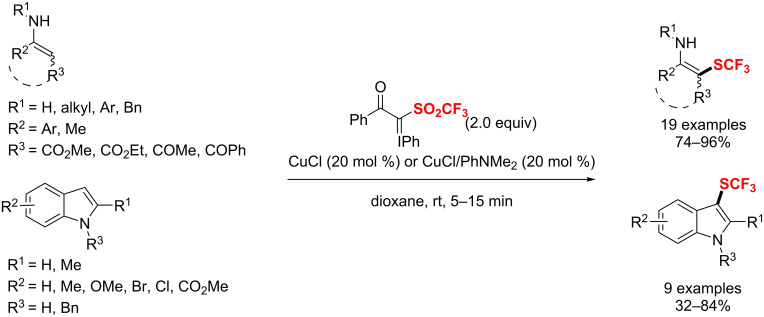
Copper(I) chloride-catalyzed trifluoromethylthiolation of enamines, indoles and β-ketoesters.

In 2013, Shibata’s group [198] developed an electrophilic trifluoromethansulfonyl hypervalent iodonium ylide for the trifluoromethylthiolation of enamines, indoles and β-ketoesters catalyzed by copper(I) chloride (Scheme 116). The desired CF_3_S-substituted products were formed with good yields in short times at room temperature.

**Scheme 117:**
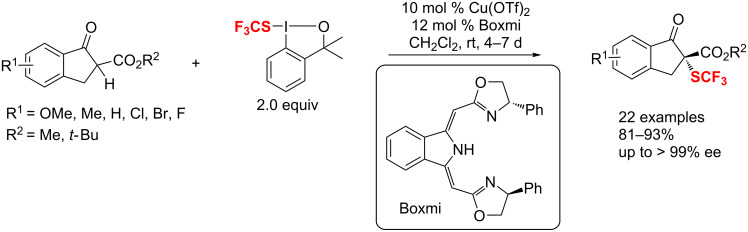
Copper-boxmi-catalyzed asymmetric trifluoromethylthiolation of β-ketoesters.

In 2014, Gade et al. [199] applied a copper-boxmi complex as highly enantioselective catalyst to effect electrophilic trifluoromethylthiolations (Scheme 117). A number of α-SCF_3_-substituted β-ketoesters were obtained in good yields with high enantiomeric excess (ee) under mild conditions.

**Scheme 118:**
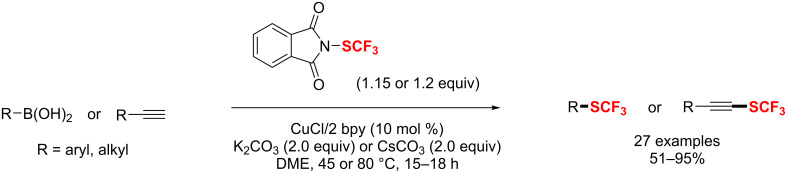
Direct Cu-catalyzed trifluoromethylthiolation of boronic acids and alkynes.

The group of Rueping [200] employed *N*-(trifluoromethylthio)phthalimide as an electrophilic source of F_3_CS^+^ for the direct trifluoromethylthiolation of boronic acids and alkynes under copper catalysis in 2014 (Scheme 118). Based on the mild conditions, this approach features high functional group tolerance and a broad substrate scope.

**Scheme 119:**
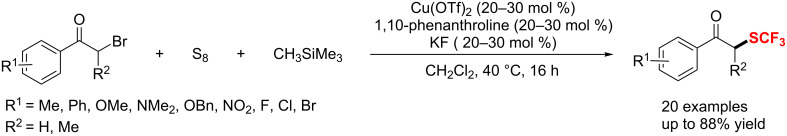
Cu-catalyzed synthesis of α-trifluoromethylthio-substituted ketones.

In the same year, a powerful protocol for the direct synthesis of α-trifluoromethylthio-substituted ketones was reported by Weng and co-workers [201]. Notably, the trifluoromethylthiolation reactions of primary and secondary α-bromoketones worked well with CF_3_SiMe_3_ and elemental sulfur as precursors (Scheme 119). Furthermore, this strategy shows a broad substrate scope and tolerates a variety of functional groups.

**Scheme 120:**
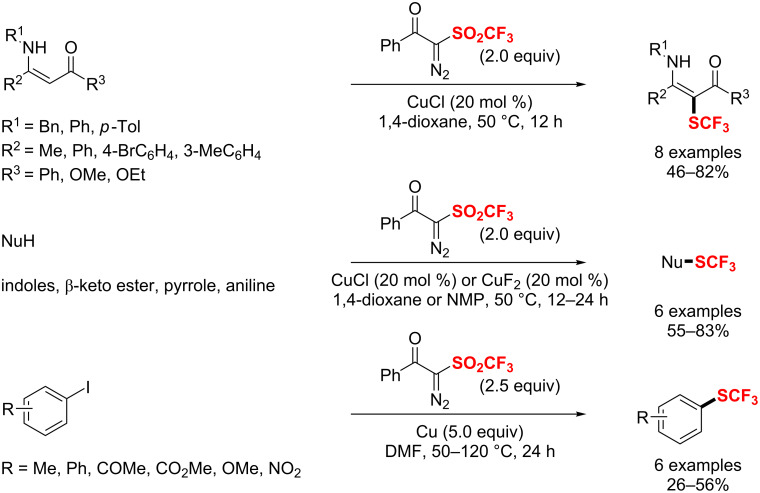
Trifluoromethylthiolation reactions promoted by diazotriflone and copper.

In 2016, a variety of enamines, indoles, β-keto esters, pyrroles, and anilines were trifluoromethylthiolated efficiently by Shibata’s group [202] in the presence of diazotriflone and copper catalysis through an electrophilic-type reaction (Scheme 120).

**Scheme 121:**
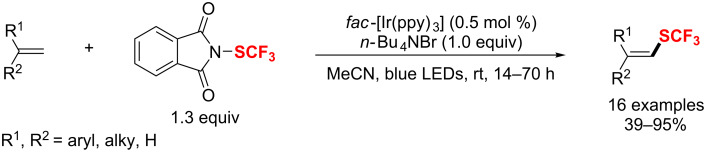
Halide activation of *N*-(trifluoromethylthio)phthalimide.

In 2016, Glorius et al. [203] introduced the synthesis of vinyl-SCF_3_ compounds using *N*-(trifluoromethylthio)phthalimide as SCF_3_ source under blue LEDs irradiation. Notably, a variety of alkenes could be converted to vinyl-SCF_3_ compounds with the cooperation of an [Ir] photocatalyst and an ammonium bromide salt (Scheme 121). The formed adduct could be triggered via an oxidative quenching cycle and delivered a SCF_3_ radical. Under similar conditions, they also demonstrated a tandem photoinduced trifluoromethylthiolation/semi-pinacol-type rearrangement of ketones.

**Scheme 122:**
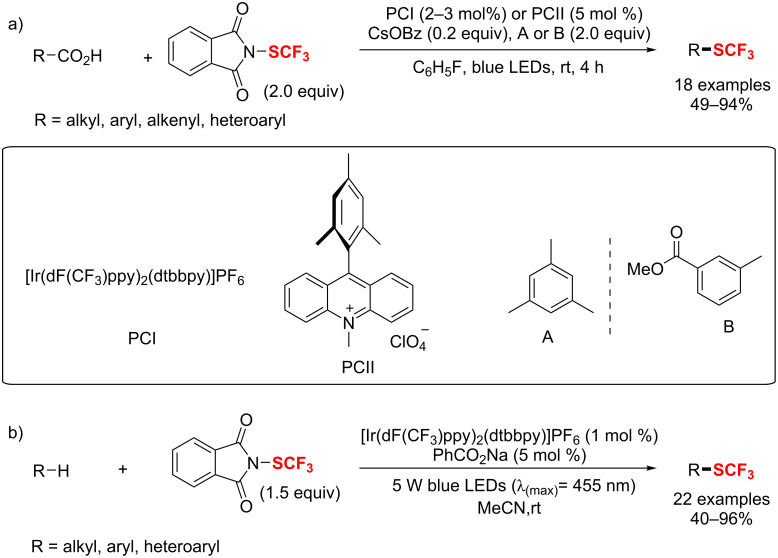
The visible light-promoted trifluoromethylthiolation reported by Glorius.

Meanwhile, the same group [204] developed a visible-light-mediated trifluoromethylthiolation of alkyl carboxylic acids with [Ir] and phtalimide-SCF_3_ as the trifluoromethylthiolating reagent (Scheme 122a). Moreover, tertiary, secondary, and primary alkyl carboxylic acids afforded the desired products in good to excellent yields. Notably, the use of an external sacrificial hydrogen atom donor, mesitylene or methyl (3-methyl)benzoate, avoided the bistrifluoromethylthiolation reaction in this process. Subsequently, the group [205] demonstrated that the Phth-SCF_3_ reagent could also be used for the direct trifluoromethylthiolation of C–H bonds under similar mild conditions (Scheme 122b). Also, a wide range of aliphatic substrates were converted to their trifluoromethylthiolated analogues in very good yields.

**Scheme 123:**
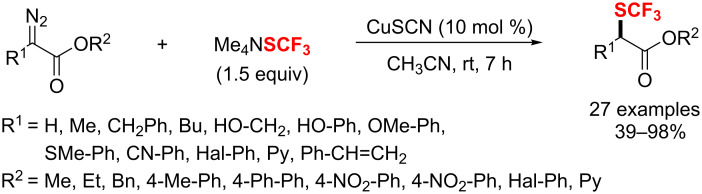
Synthesis of α-trifluoromethylthioesters via Goossen’s approach.

Additionally, the group of Goossen [206] disclosed a simple and practical strategy for the conversion of α-diazo esters to the corresponding trifluoromethylthiolated esters with a Me_4_NSCF_3_ salt. In the presence of copper thiocyanate, this transformation afforded the products with up to 98% yields at room temperature and was applied to a wide range of easily available α-diazo esters (Scheme 123).

**Scheme 124:**
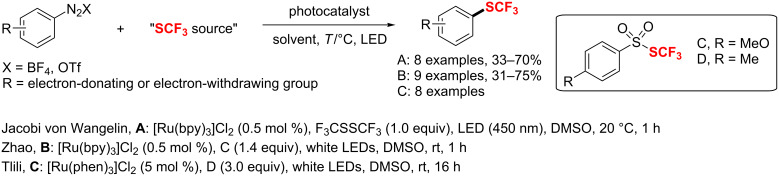
Photoinduced trifluoromethylthiolation of diazonium salts.

Recently, the formation of arenes-SCF_3_ was shown by the Jacobi von Wangelin group [207], the Zhao group [208] as well as the Tlili group [193] (Scheme 124). All three methodologies have been developed based on a [Ru]-based photocatalyst under LED irradiation. The group of Jacobi von Wangelin used the bis(trifluoromethyl) disulfide (CF_3_SSCF_3_) as the source of trifluoromethyl sulfide, while the other two groups employed shelf-stable reagents **C** and **D** for trifluoromethylthiolation, arenesulfonate-SCF_3_.

### Trifluoromethoxylation

The introduction of a trifluoromethoxy (OCF_3_) group into a molecule can improve its metabolic stability and membrane permeability. Some strategies for the synthesis of trifluoromethoxylated compounds have been reviewed: Poisson [209] and Billard [210] discussed the recent advances toward the synthesis of OCF_3_-containing molecules; Hopkinson [211] depicted a radical revolution for trifluoromethoxylation; recently, Ngai [212] summarized some photoredox-based approaches to form tri- and difluoromethoxylated compounds. Despite the great interest in this functional group, only a few transition-metal-catalyzed methods have been developed for the synthesis of trifluoromethoxylated compounds over the past decade. This may be due to the fact that C–OCF_3_ bond formation reactions have many limitations, including reversible decomposition of the trifluoromethoxide anion in solution above room temperature to afford carbonic difluoride and fluoride [213–214], as well as β-fluoride elimination from transition-metal-trifluoromethoxide complexes [215–216].

**Scheme 125:**
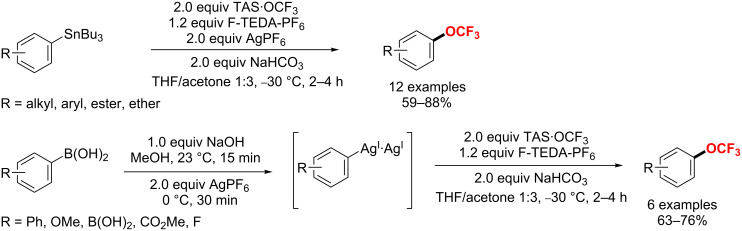
Ag-mediated trifluoromethoxylation of aryl stannanes and arylboronic acids.

In 2011, the first report of a transition-metal-mediated C_aryl_–OCF_3_ bond formation was described by the Ritter group (Scheme 125) [217]. Aryl trifluoromethyl ethers could be accessed through a silver-mediated cross-coupling of trifluoromethoxide with arylstannanes and arylboronic acids.

**Scheme 126:**
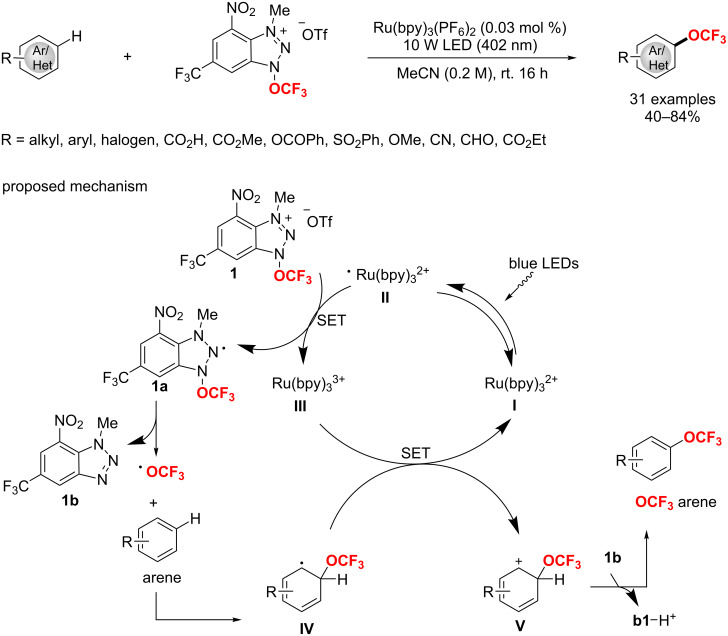
Catalytic (hetero)aryl C–H trifluoromethoxylation under visible light.

In 2018, Ngai and co-workers [218] reported a direct C–H trifluoromethoxylation of (hetero)arenes under visible light irradiation. This approach proceeded at room temperature by employing the redoxactive catalyst Ru(bpy)_3_(PF_6_)_2_ (Scheme 126). Mechanism studies suggest a SET from the excited photoredox catalyst to **1** resulting in exclusive liberation of the OCF_3_ radical. The reaction of the trifluoromethoxyl radical with (hetero)arenes provides trifluoromethoxylated cyclohexadienyl radicals that undergo oxidation and deprotonation to generate the desired products.

**Scheme 127:**
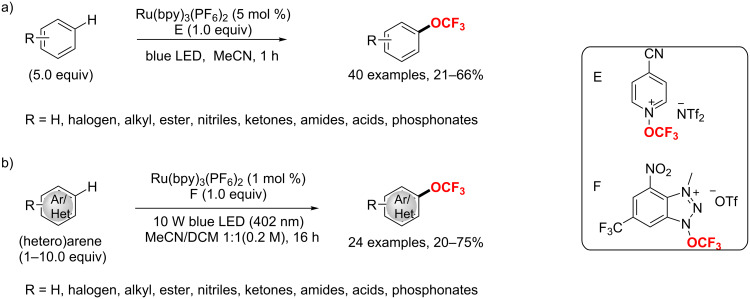
Photoinduced C–H-bond trifluromethoxylation of (hetero)arenes.

Recently, Ngai’s group [219] as well as the Togni group [220] independently developed cationic N–OCF_3_ reagents. Both reagents are reducible with an excited ruthenium-based photocatalyst. Herein, Togni synthesized a series of *N*-trifluoromethoxypyridinium reagents (Scheme 127a), while Ngai prepared a series of 1-CF_3_O-benzotriazole reagents (Scheme 127b). These reagents exhibit the best results for the direct C–H trifluoromethoxylation of (hetero)arenes. Notably, both groups used the same photocatalyst ([Ru(bpy)_3_](PF_6_)_2_) under blue LED irradiation. Of note, the cationic N–OCF_3_ reagents presented an impressive substrate scope tolerance including halides (I, Br, Cl and F), nitriles, ketones, amides, acids and phosphonates.

## Conclusion

The development of methods for the transition-metal-catalyzed incorporation of fluorine-containing groups into target molecules is an active area of chemical research. In this review, we summarized the major advances in the field of transition-metal-catalyzed fluorination and fluoroalkylation reactions over the past few years. A variety of methods shows significant advantages in view of atom-economy, reaction diversity, selectivity and functional-group compatibility. The suitable catalytic systems and the newly developed reagents play a critical role in these reactions. Further, different directing groups also contribute greatly to the success of these reactions. Notably, copper shows a wider range of applications involving the catalysis of fluorination/fluoroalkylation reactions of various alkyl-, aryl- and vinyl- as well as alkynyl substrates, while palladium-catalyzed reactions in combination with suitable ligands show improved selectivity in some reactions. Also, because of milder and cleaner reaction conditions, photocatalysts have received extensive attention and have greatly applied recently. Despite diverse methods for the transition-metal-catalyzed fluorination/fluoroalkylation have been reported, chemists still face many challenges that need to be overcome. More economical, green, selective, general and practical strategies remain sought after. A suitable catalytic system and an effective fluorination reagent are two important aspects for achieving this goal. Overall, we hope that this review will provide further insight into this field and inspire chemists to develop new fluorination/fluoroalkylation reactions. Meanwhile, we believe that the methodologies mentioned in this review will contribute to future advances in the late-stage functionalization of molecules.
